# Structure of the Golgi apparatus is not influenced by a GAG deletion mutation in the dystonia-associated gene *Tor1a*

**DOI:** 10.1371/journal.pone.0206123

**Published:** 2018-11-07

**Authors:** Sara B. Mitchell, Sadahiro Iwabuchi, Hiroyuki Kawano, Tsun Ming Tom Yuen, Jin-Young Koh, K. W. David Ho, N. Charles Harata

**Affiliations:** 1 Department of Molecular Physiology and Biophysics, University of Iowa Carver College of Medicine, Iowa City, Iowa, United States of America; 2 Department of Chemical and Biochemical Engineering, University of Iowa College of Engineering, Iowa City, Iowa, United States of America; 3 Medical Scientist Training Program, University of Iowa Carver College of Medicine, Iowa City, Iowa, United States of America; 4 Iowa Neuroscience Institute, University of Iowa Carver College of Medicine, Iowa City, Iowa, United States of America; Hertie Institute for Clinical Brain Research and German Center for Neurodegenerative Diseases, GERMANY

## Abstract

Autosomal-dominant, early-onset DYT1 dystonia is associated with an in-frame deletion of a glutamic acid codon (ΔE) in the *TOR1A* gene. The gene product, torsinA, is an evolutionarily conserved AAA+ ATPase. The fact that constitutive secretion from patient fibroblasts is suppressed indicates that the ΔE-torsinA protein influences the cellular secretory machinery. However, which component is affected remains unclear. Prompted by recent reports that abnormal protein trafficking through the Golgi apparatus, the major protein-sorting center of the secretory pathway, is sometimes associated with a morphological change in the Golgi, we evaluated the influence of ΔE-torsinA on this organelle. Specifically, we examined its structure by confocal microscopy, in cultures of striatal, cerebral cortical and hippocampal neurons obtained from wild-type, heterozygous and homozygous ΔE-torsinA knock-in mice. In live neurons, the Golgi was assessed following uptake of a fluorescent ceramide analog, and in fixed neurons it was analyzed by immuno-fluorescence staining for the Golgi-marker GM130. Neither staining method indicated genotype-specific differences in the size, staining intensity, shape or localization of the Golgi. Moreover, no genotype-specific difference was observed as the neurons matured *in vitro*. These results were supported by a lack of genotype-specific differences in GM130 expression levels, as assessed by Western blotting. The Golgi was also disrupted by treatment with brefeldin A, but no genotype-specific differences were found in the immuno-fluorescence staining intensity of GM130. Overall, our results demonstrate that the ΔE-torsinA protein does not drastically influence Golgi morphology in neurons, irrespective of genotype, brain region (among those tested), or maturation stage in culture. While it remains possible that functional changes in the Golgi exist, our findings imply that any such changes are not severe enough to influence its morphology to a degree detectable by light microscopy.

## Introduction

The Golgi apparatus is an intracellular, membrane-bounded organelle, and it is involved in protein-lipid modification as well as in trafficking of transport vesicles from the endoplasmic reticulum (ER) to the plasma membrane [1–4]. The Golgi is increasingly recognized as an organizing center for microtubules [5, 6], indicating that its function can affect a broad spectrum of trafficking phenomena along the microtubules. In neurons, it resides mainly in the soma, near the nucleus (paranuclear Golgi apparatus) [7], where it is crucial for the transport of specific proteins to dendrites and axons [8–10]. The Golgi is also present in the proximal dendrite (dendritic Golgi outpost), where it controls dendrite growth and neuron polarity [11, 12]. Thus the Golgi apparatus is important in regulating the structure and functions of neurons, and it is not surprising that abnormalities in Golgi function have been associated with neurological disorders [10, 13, 14].

DYT1 dystonia is a developmental neurological disorder that affects the brain [15–17] and was recently classified as a part of "early-onset generalized isolated dystonia" [18]. It is one of the most common inherited forms of dystonia, is autosomal-dominant, and is characterized by sustained contraction of muscles in the limbs and trunk, resulting in a twisted posture. The pathophysiology of DYT1 dystonia is thought to arise from abnormalities in synaptic transmission, neuronal microcircuitry and/or large-scale neural networks [19–23]. This notion is based on clinical findings of enhanced neural-network excitability in patients (e.g. [24]), and on a lack of evidence for neurodegeneration, inflammation or other major macroscopic changes in the brain [25–27]. DYT1 dystonia is caused by a mutation in the *TOR1A* gene (c.904_906delGAG/907_909delGAG; p.Glu302del/Glu303del; *TOR1A*^ΔE^), which encodes the protein torsinA; this mutation results in an in-frame deletion of a codon for glutamic acid (ΔE-torsinA) [28, 29]. TorsinA belongs to the ATPases associated with diverse cellular activities (AAA+) family of proteins, whose members generally perform chaperone-like functions, assisting in protein unfolding, protein-complex disassembly, membrane trafficking, and membrane fusion [30–32]. Its homologs are evolutionarily conserved and found in species ranging from *C*. *elegans* to humans [33–35]. However, the details of how ΔE-torsinA influences the mammalian central neurons are still not well understood.

At least three sets of data are indirectly consistent with structure of the Golgi apparatus being affected in DYT1 dystonia. First, the constitutive secretion of an artificial reporter (*Gaussia* luciferase) was suppressed in cultured fibroblasts from DYT1 patients [36, 37]. This result suggests that some component of the secretory pathway, from the ER through the Golgi to the plasma membrane, is affected, although the Golgi abnormality was not examined specifically. Second, in dystonia musculorum in mice (that corresponds to the hereditary sensory autonomic neuropathy type VI [HSAN-VI] in humans [38]), mutations in the dystonin (*DST*) gene disrupt the organization of the Golgi apparatus, stability of the microtubules, and transport of secretory molecules through the cell [39, 40]. This disease phenotype is interesting in that it potentially links distal contractures (dystonia) with a Golgi abnormality, although dystonia musculorum differs from DYT1 in that it is a neurodegenerative disease and is characterized by progressive ataxia as well as prominent degeneration of the dorsal root ganglia neurons [41–43], and thus differs from human dystonia. Third, changes in the shape of the Golgi have been identified as early signs of neurological disorders; for example, Parkinson's disease [44, 45], which is associated with an accumulation of the pre-synaptic protein α-synuclein and a block in ER-Golgi transport [46].

These lines of circumstantial evidence led us to hypothesize that morphology of the Golgi apparatus could be compromised in neurons with a *Tor1a*^ΔE^ mutation. Since the pathophysiology of DYT1 dystonia is not well understood, any insights into cellular manifestations would be important. If a morphological abnormality were detectable, it might be possible to use it as a disease marker, and could facilitate the screening of potential treatment options, given the lack of clear morphological phenotypes in neuron-specific organelles at the light microscopic level. Notably, the Golgi apparatus had not been analyzed for changes in morphology.

We tested our hypothesis by examining the morphology of Golgi apparatus in cultured CNS neurons that bear the *Tor1a*^ΔE^ mutation. We used two approaches to stain the Golgi. In one, we analyzed live neurons based on cellular uptake of a fluorescent ceramide analogue and its accumulation in the Golgi apparatus [47–49]. In another, we used fixed-cell immunocytochemistry for Golgi matrix protein of 130 kDa (GM130), a well-established marker of the *cis*-Golgi [11, 50]. GM130 is key to several processes, including fusion between transport vesicles originating at the ER and Golgi membranes [51], nucleation of microtubules on the Golgi [6], spindle assembly and cell division [52], and regulation of the compartmental organization of dendritic Golgi outposts [53]. Indeed, mutation in the GM130-encoding gene (*GOLGA2*) leads to a human neurological disorder [54]. We examined the stained Golgi apparatus by light microscopy, and compared its size, intensity and shape, as well as the localization within the soma of mutant and wild-type neurons. We also compared the percentage of neurons with Golgi outposts and their lengths, and assessed mutant neurons for effects on the size of the Golgi over the course of their culture.

Cultured neurons were obtained from mice of the ΔE-torsinA knock-in model, in which the endogenous mouse *Tor1a* gene harbors the mutation that is found in human patients (*TOR1A*^+/ΔE^) [55, 56]. This better reflects the situation in human patients than either overexpression of the mutant transgene or knock-out of the wild-type gene. We imaged neurons of the cerebral cortex, striatum and hippocampus as described previously [57–62]. The cell-culture system is advantageous, because it allows us to use homozygous knock-in mice which do not survive to adulthood [55, 56, 63], and it enables imaging at high spatial resolution with minimal interference from adjacent regions.

We report that, contrary to our hypothesis, the *Tor1a*^ΔE^ mutation did not significantly influence the morphology of the Golgi apparatus in neurons of any of the three brain regions, any of the two genotypes, or any of the culture stages tested (from day 11 to 26).

## Materials and methods

### Ethics statement

Animal care and use procedures were approved by the University of Iowa's Institutional Animal Care and Use Committee (Protocol number: 7021969), and performed in accordance with the standards set by the PHS Policy and The Guide for the Care and Use of Laboratory Animals (NRC Publications) revised 2011. Every effort was made to minimize suffering of the animals.

### Genotyping

On postnatal days 0–1, pups of the ΔE-torsinA knock-in mouse model of DYT1 dystonia [55], of both sexes, were genotyped and identified as wild-type (WT, *Tor1a*^+/+^), heterozygous (HET, *Tor1a*^+/ΔE^) or homozygous (HOM, *Tor1a*^ΔE/ΔE^) mice, using a fast-genotyping procedure (completed within 4 hours, EZ Fast Tissue/Tail PCR Genotyping Kit, EZ BioResearch LLC, St, Louis, MO) [62].

### Cell culture

Neurons from individual newborn pups were cultured separately by a method described previously [62]. In brief, the striatum, cerebral cortex and hippocampus were dissected at postnatal days 0–1. In removing the striatal samples, we took only the region dorsal to the anterior commissure so that the nucleus accumbens was avoided as much as possible. Samples of cerebral cortex were taken from the region immediately dorsal to the striatum [64], which includes the motor cortex [65]. The hippocampus included the CA3-CA1 region, but excluded the dentate gyrus [57, 66]. The dissected tissues were trypsinized and mechanically dissociated. The cells were plated on 12-mm coverslips (thickness No. 0, Carolina Biological Supply, Burlington, NC) previously seeded with a rat hippocampal glial feeder layer [67], in 24-well plates. Cells isolated from the striatum, cerebral cortex and hippocampus of each pup were plated at densities of 24,000, 10,000 and 12,000 cells per well, respectively. The cells were cultured in a humidified incubator at 37°C, with 5% CO_2_. The cultured neurons were analyzed after 11–26 days *in vitro* (DIV). For each genotype, the experimental data were obtained from 3–7 separate culture batches (i.e., pups).

### Golgi staining in live cells

The Golgi apparatus in live neurons was stained using a fluorescent ceramide analog, N-(4,4-difluoro-5,7-dimethyl-4-bora-3a,4a-diaza-s-indacene-3-pentanoyl)sphingosine (BODIPY FL C_5_-ceramide) (B-22650; Thermo Fisher Scientific, Waltham, MA), which is equivalent to the "C_5_-DMB-ceramide" first reported in [47], after being complexed to bovine serum albumin (BSA) at 1:1 molar ratio in order to reduce hydrophobicity.

Staining was carried out according to the original report [47] and the vendor information, with slight modifications. Cultured neurons were incubated with 5 μM BODIPY-FL C_5_ ceramide-BSA in Minimum Essential Media (MEM, Thermo Fisher Scientific) at 4°C (on ice) for 30 min. Phase-contrast microscopy revealed that cell viability was not affected by incubation at 4°C instead of room temperature. The cells were then gently washed, by aspirating the existing solution and pouring in fresh solution before exposing the cells to air (pull-push method), three times each with 700 μl MEM at 4°C. The coverslips were transferred to pre-warmed MEM (kept at 37°C in the incubator for at least 1 hr). After the cells were stained at 37°C for 30 min in the incubator, they were washed by the pull-push method at room temperature, 3–6 times each with 700 μl Tyrode's solution (containing, in mM: 125 NaCl, 2 KCl, 2 CaCl_2_, 2 MgCl_2_, 30 D-glucose, 25 HEPES, pH 7.4 adjusted with 5 M NaOH, ~310 mOsm without adjustment). The cells were transferred to an imaging chamber (RC-26, Warner Instruments, Hamden, CT), which has a glass floor of thickness No. 0 (72198–21, Electron Microscopy Sciences, Hatfield, PA). Live cells were washed for 5–10 min before imaging, and the imaging chamber was constantly bath-perfused with fresh Tyrode's solution during imaging, as in physiology experiments on live cells [68].

We tested other Golgi probes: NBD C_6_-Ceramide (Thermo Fisher Scientific), which is equivalent to the "C_6_-NBD-ceramide" reported in [69], and BODIPY TR ceramide (Thermo Fisher Scientific). However, these reagents led to high background staining in the cytoplasm, and were thus not used in the current study.

In some live neurons, the nuclei were counterstained using membrane-permeant Hoechst 33342 (H1399, Thermo Fisher Scientific). After the Golgi staining (after washing 3–6 times with 700 μl Tyrode's solution), the neurons were treated with the dye at 0.5 μg/ml in Tyrode's solution for 10 min at room temperature, and washed for 3–5 min with Tyrode's solution. Then the neurons were transferred to the imaging chamber.

In the present study, the "Golgi staining" refers to the staining of the Golgi apparatus, not to be confused with the Camillo Golgi's silver-impregnation staining of neurons.

### Golgi staining in fixed cells

GM130 was stained using our immunocytochemistry protocol [60] with slight modifications. The cultured neurons were fixed with 4% paraformaldehyde (15710, Electron Microscopy Sciences) and 4% sucrose in Tyrode's solution for 30 min at 4°C. After being washed with Tyrode's solution twice (5 min per wash with the pull-push method) at 4°C, the cells were permeabilized with 0.1% Triton X-100 in Tyrode's solution for 10 min, and washed with Tyrode's solution three times (5 min per wash). They were blocked with 2% normal goat serum (G-9023, Sigma-Aldrich, St. Louis, MO) in phosphate-buffered saline (PBS, 70011–044, pH 7.4, Thermo Fisher Scientific) (blocking solution), for 60 min at room temperature. Thereafter they were treated with mouse monoclonal anti-GM130 antibody (610822, BD Biosciences, San Jose, CA) (400*×* dilution in blocking solution) overnight (15–21 hours) at 4°C. Following washing with PBS 3 times (7 min per wash), the neurons were incubated with goat anti-mouse IgG antibody conjugated with Qdot 605 (Q11002MP, Thermo Fisher Scientific) (50*×* dilution in blocking solution) for 60 min at room temperature. They were washed with PBS at least 5 times (20 min per wash), transferred to the imaging chamber, and observed directly in PBS.

Co-staining for GM130 and the dendritic marker microtubule-associated protein 2 (MAP2) were performed according to a protocol similar to that described above, with the following changes. The fixed cells were treated with the anti-GM130 antibody and the rabbit polyclonal anti-MAP2 antibody (AB5622, MilliporeSigma, Billerica, MA) (mixed at 400*×* dilution in blocking solution) for 42–48 hours. The secondary antibodies were goat anti-mouse IgG antibody conjugated with Alexa Fluor 488 (A-11001, Thermo Fisher Scientific) and goat anti-rabbit IgG antibody conjugated with Alexa Fluor 568 (A-11011, Thermo Fisher Scientific) (1,000*×* dilution in blocking solution). After being washed in PBS following incubation with the secondary antibodies, the coverslips were mounted using ProLong Diamond Antifade Mountant with DAPI (P36962, Thermo Fisher Scientific). They were cured at room temperature for a minimum of 24 hours, and a minimum of 24 hours at 4°C.

### Golgi disruption

Cultured neurons that had been grown on coverslips were treated with either 1 μg/ml brefeldin A (1:10,000 dilution of a 10-mg/ml stock solution in dimethyl sulfoxide (DMSO); B5936, Sigma-Aldrich) or vehicle at the same concentration (DMSO diluted 1:10,000; D2438, Sigma-Aldrich). The brefeldin A stock solution and DMSO were diluted in two steps. First, each was diluted 1:100 in 1-ml MEM that had been pre-warmed overnight. Second, each diluted solution was added directly and gently to wells containing the cultured neurons and 1 ml of culture medium, resulting in dilution of the first solution 100 times. The neurons were left in these solutions for 2 hours at 37°C, in a culture incubator kept at 5% CO_2_. After these treatments, the coverslips were transferred into other wells containing 1 ml Tyrode's solution and kept therein for several minutes. Thereafter the neurons were chemically fixed and stained for GM130, MAP2 and DAPI as described above.

### Cell imaging

In order to avoid bias in the acquisition of fluorescence images, neurons were chosen for imaging based on transmitted light optics, i.e. differential interference contrast (DIC) or phase-contrast microscopy, without prior information about the fluorescence staining. The selected neurons displayed normal morphological features (such as a clear somatic margin), and did not display signs of deterioration (such as the beaded appearance of dendrites and the formation of spherical intracellular vacuoles in the surrounding glial cells). For quantitative analysis of the Golgi, imaging was restricted to neurons whose somata and proximal dendrites were not in direct contact with other neurons. All imaging experiments were performed at room temperature (23–25°C).

### Fluorescence imaging system

**Fig 1:**
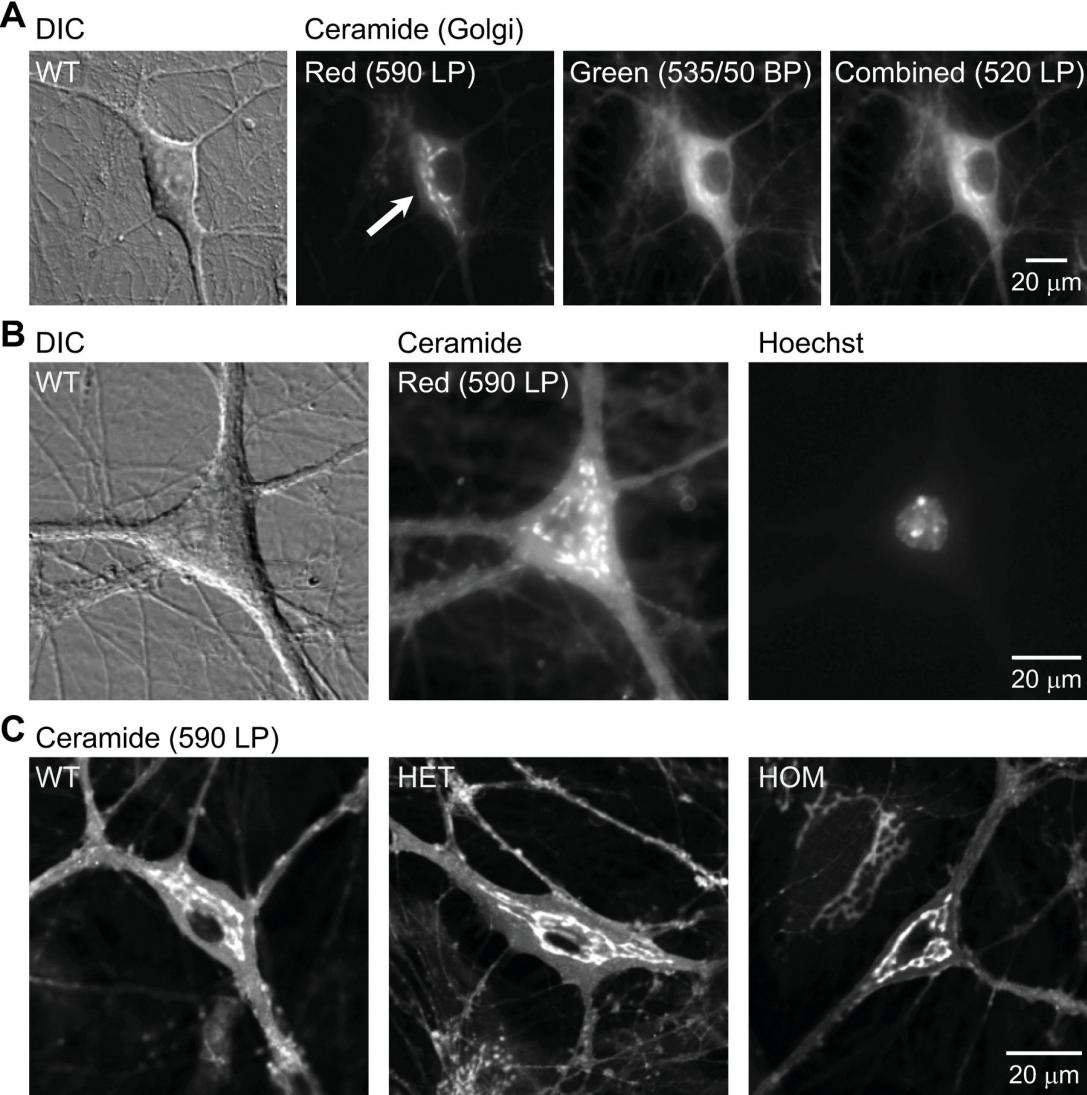
Live-cell staining of the Golgi apparatus by uptake of a fluorescent ceramide analogue. Imaging was carried out in hippocampal cultures at 15–19 days *in vitro* (DIV). **A:** Differential interference contrast (DIC) and widefield fluorescence microscopy images of a cultured neuron. It was obtained from a wild-type (WT) mouse and stained with BODIPY FL C_5_-ceramide (Ceramide). The dye was excited by blue light, and the emitted light was observed using the red long-pass (590-nm LP), green band-pass (535/50-nm BP), and green long-pass (520-nm LP) emission filters. Imaging with the red emission filter produced the strongest contrast between Golgi (specific) and ER (non-specific) staining (arrow). The image contrast was adjusted, such that the maximal and minimal intensities in each image were the same. **B:** DIC and widefield fluorescence microscopy images of BODIPY FL C_5_-ceramide-stained WT neuron. The nucleus was stained with Hoechst dye. Ceramide signal was visualized using the red emission filter. **C:** Confocal fluorescence microscopy image of BODIPY FL C_5_-ceramide-stained neurons obtained from WT, heterozygous (HET) and homozygous (HOM) ΔE-torsinA knock-in mice. Fluorescence imaging was carried out using the red filter.

For general characterization of the Golgi apparatus by widefield optics, the neurons were imaged using an inverted microscope (Eclipse-TiE, Nikon, Melville, NY) equipped with an interline CCD camera (Clara, Andor Technology, Belfast, UK). The camera was cooled at -45°C with the aid of an internal fan. BODIPY FL C_5_-ceramide was excited using a 490-nm light-emitting diode (LED, CoolLED, Andover, UK) at 100% intensity, and imaged using a filter cube, 490/20-nm excitation (EX), 510-nm dichroic-long-pass (DCLP), 590-nm-long-pass emission (EM) ("red"), and 2-sec exposure. In the experiments described in Fig 1, the signal was also imaged using an emitter filter of 535/50-nm-band-pass emission ("green") or 520-nm-long-pass emission ("combined"), and 2-sec exposure. Hoechst 33342 was excited using a 400-nm LED (CoolLED) at 100% intensity, and imaged with a filter cube (405/40-nm EX, 440-nm DCLP, 470/40-nm EM), and 0.2-sec exposure. 16-bit images were acquired using a 40*×* objective lens (Plan Fluor, numerical aperture 1.30, Nikon) with a coupler (0.7*×*) and without binning, in the single-image capturing mode of the Solis software (Andor).

For quantitative characterization of the Golgi apparatus, the neurons were imaged using a confocal laser-scanning microscope (510 confocal, Carl Zeiss MicroImaging GmbH, Göttingen, Germany) equipped with an inverted microscope (Axiovert 100M, Carl Zeiss) and a 40*×* objective lens (Plan-Neofluor, numerical aperture 1.30). BODIPY FL C_5_-ceramide and Qdot 605 were excited using the 488-nm line of an argon laser at 10% intensity, and imaged using a 585-nm-long-pass emission. Voxel sizes were 0.22 *×* 0.22 *×* 0.44 μm. Pinhole size was 58 μm with an Airy unit of 0.8. Pixel time was 1.6 μs. Four frames were averaged to give a single 12-bit image at each level of focus (optical section), and a stack of images (z-stack) was acquired at levels 0.44-μm apart. In some live-imaging experiments, the Golgi apparatus was visualized using another confocal microscope (SP8, Leica Microsystems, Buffalo Grove, IL). The same imaging conditions were used, including the objective lens properties, excitation and emission wavelengths, voxel sizes and the number of frames averaged.

For the analysis of dendritic Golgi outposts or disrupted Golgi in a large number of fixed neurons, images were acquired from an entire coverslip surface using a slide scanner (VS120-S5 Virtual Slide Microscope; Olympus, Center Valley, PA). We used a 20*×* air lens at a measured resolution of 0.32 μm/pixel, and the exposure time was 50 ms (DAPI channel), 300 ms (FITC channel) and 100 ms (TRITC channel) with a filter set (DA/FI/TR/Cy5-4X4M-C-000; Semrock, Rochester, NY).

Imaging conditions were kept the same for each brain region of the same culture batch, such that the intensities can be compared across neurons.

### Cellular image analysis

Cellular images were analyzed using the ImageJ software (version 1.51r, W. S. Rasband, National Institutes of Health, Bethesda, MD) and MATLAB (version 9.2, MathWorks, Natick, MA).

For measurements of the area and staining intensity of the Golgi apparatus using confocal microscopy, multiple regions of interest (ROIs) of arbitrary shapes were manually assigned within the diffusely and uniformly stained background of the somatic cytoplasm in an image (excluding nucleoplasm). An intensity histogram was created from all the pixels within the ROIs (total of >3,000 pixels) using Microsoft Excel (Redmond, WA). The maximal intensity within these regions was chosen as the intensity threshold, and was used to select all pixels in the same image whose intensity was larger than the threshold value (thresholding). The number and intensity of the selected pixels were used to calculate the area (in μm^2^) and the average pixel staining intensity of the Golgi apparatus. In order to minimize the effect of size variation across the same Golgi at different focus levels, we measured the above parameters in one of two ways: the average of values measured in three consecutive images (3-image-average method); or a set of values from a single image created by projecting images in a z-stack using the maximum intensity projection (MIP) algorithm. The used method is described for each analysis.

The somatic area was measured by manually tracing the area within the background fluorescence level. This included both the cytoplasm and the nucleoplasm.

The length of a dendritic Golgi outpost was defined as the distance from its distal tip to the dendrite-soma border. If a neuron had more than one outpost, the length of the longest of these was measured. Dendritic outposts were included in almost all analyses of this study; the single exception was calculation of the Golgi fraction of the cellular area (Golgi area / somatic area) in mature neurons (>16 DIV). In this case, dendritic outposts were excluded and only the somatic Golgi was included in the calculation.

To determine the extent to which the Golgi surrounded the nucleus, we measured the angle subtended by the edges of the Golgi apparatus (encompassing the widest area of Golgi) in the confocal images of ceramide staining, using the center of the nucleus as the vertex. For each neuron, three consecutive optical sections were chosen to represent the thickest area of nucleus (identified as the darkest region within a neuronal soma), and their maximal intensity projection was obtained. When a spatial gap existed within the Golgi (found in ~5–10% of neurons), it was ignored (i.e., the angle was measured as if the gap did not exist). Note that this analysis does not represent the three-dimensional structural information. However, to minimize error, we excluded neurons in which the bulk of the Golgi body was situated above or below the planes of three nuclear sections.

We also assessed the extent to which the Golgi was concentrated (polarized) at the base of the thickest dendrite, using slide-scanner images and a method similar to the one published previously [11]. Briefly, MAP2 staining was used to align the somatic centers of all neurons (based on manual tracing and determining the center of mass without weighting by intensity) and to orient them with the thickest dendrite pointing in the 12 o'clock direction. The GM130 images of those neurons (irrespective of whether they had dendritic outposts) were then cropped to 100 *×* 100 pixels, and averaged after the maximum intensity in each 16-bit image was normalized to an arbitrary value of 65535 = 2^16^–1. The relative distribution of the Golgi was analyzed as the averaged pixel intensities in quadrants (90° pies) of a circle inscribed in the square image, with the top quadrant encompassing the 12-o'clock direction. Our method was adapted from the published protocol [11], differing from it in two respects. First, the dendrite with the thickest base, rather than the one that was longest, was analyzed, because this approach minimizes contamination from the dendrites of neighboring neurons. Second, the intensity in each image was normalized to enable comparisons of the spatial distribution of fluorescence signals across genotypes, and thus to eliminate any subtle differences in fluorescence intensity between HET and WT neurons even if it existed.

To determine the extent to which the Golgi was disrupted by brefeldin A treatment, we measured the staining intensity of GM130 in slide-scanner images. For this purpose, we subtracted background noise using a rolling-ball method (radius of 7 pixels) [70], and measured the average intensity of all pixels whose intensities were above the threshold value determined by the auto-thresholding function of ImageJ.

### Western blotting

After the culture medium was removed, cells cultured on a 12-mm round coverslip were lysed and the proteins were solubilized in 75 μl of lysis buffer (50 mM Tris-HCl, 150 mM NaCl, 5 mM EDTA, pH 7.4, 1% SDS). After the lysates were heated to 70°C for 10 min, the volumes of lysates were adjusted (see below), and the diluted samples containing equal amounts of total protein were subjected to SDS-PAGE (4%-12% gradient precast gels) (Invitrogen) under reducing conditions in triplicate, and then transferred onto a polyvinylidene difluoride (PVDF) membrane. Membranes were incubated in Tris-buffered saline containing 0.1% Tween 20 (TBST) and 5% nonfat milk (w/v) for 1 h, and then in TBST containing 5% bovine serum and at a final concentration of 0.5 μg/ml (1:500 dilution) of the mouse monoclonal anti-GM130 antibody overnight at 4°C. The blots were washed 3 times (5 min per wash) in TBST, and then incubated with a goat anti-mouse immunoglobulin IgG conjugated with horseradish peroxidase (172–1011, Bio-Rad Laboratories, Hercules, CA) at 1:10,000 dilution in TBST, for 1 h at room temperature. The blots were washed 3 times (5 min per wash) in TBST, and immunoreactive bands were developed using the ECL reagent kit (SuperSignal West Femto Chemiluminescent Substrate, 34094, Thermo Fisher Scientific, Waltham, MA). The blots were exposed to Hyperfilm ECL film (248300, Research Products International, Mt. Prospect, IL).

The total amount of protein in each sample was used as a loading control [71–73]. Protein amounts were determined and adjusted in three steps. First, an internal control was prepared by pooling the lysates from ~20 coverslips of similarly cultured cells. Its protein concentration was measured using the bicinchoninic acid (BCA) Protein Assay Reagent Kit (23227, Thermo Fisher Scientific), with bovine serum albumin at serial dilutions serving as a standard. Second, the silver staining was used to determine the protein concentrations of un-diluted samples from single coverslips. The samples were run in duplicate and with serial dilutions of the internal control (to ensure that measurements based on optical density were linear) [72]. Total protein in each lane was measured based on the total optical density of an entire lane (vertical strip) after subtracting the background level outside the lane [71]. Third, the volume of un-diluted sample for the Western blotting was calculated, based on the results of the second step. It was confirmed with a second silver staining, by applying the calculated volumes of samples in triplicate.

To quantify the optical density of an entire lane, we used the basic rectangular measurement tool of ImageJ. We did not use built-in functions (such as Analyze\Gels) because they have a band-enhancing feature that is optimized for measuring the optical density of individual bands. This feature would underestimate the total density by treating the continuous signals between optical density peaks (bands) as background noise and subtracting them, similarly as in background-subtraction algorithms based on the spatial frequency of cellular imaging data [70].

### Antibody characterization

Anti-GM130 is a mouse monoclonal antibody of the IgG1,κ isotype, and was raised against amino acids 869–982 of rat GM130. According to the manufacturer, it recognizes a single species of approximately 130 kDa on Western blots of rat-brain homogenates. The immunocytochemical labeling pattern in the current study was the same as that observed in previous reports in which the Golgi apparatus was labeled in cultured cells, including HeLa cells [74, 75], rat hippocampal neurons [11, 76], and mouse cerebral cortex neurons [77], as well as neurons *in vivo*, in tissues such as the rat hippocampus [11, 78], rat cerebral cortex [11], mouse hippocampus [79] and mouse cerebral cortex [10]. The labeling was confirmed by immuno-electron microscopy of neurons *in vivo*, in tissues including the rat hippocampus and cerebral cortex [11], and the mouse cerebral cortex [10].

Anti-MAP2 is a rabbit polyclonal antibody raised against the protein purified from rat brain. This antibody was used to immunocytochemically stain cultured, mouse hippocampal [80] and human neurons [81].

As expected, when the primary antibodies were omitted from the immunocytochemical procedures in each experiment but the same imaging conditions and contrast were used, no signal was observed (data not shown).

### Drugs

All chemical reagents were purchased from Sigma-Aldrich unless otherwise specified.

### Statistical analyses

Measured values were compared using the unpaired, two-tailed Student's *t*-test or chi-squared test.

## Results

### Live-cell staining of the Golgi apparatus in cultured hippocampal neurons

The overall structure and position of the neuronal Golgi apparatus were assessed in live cells. We used cultured hippocampal neurons for this analysis because this brain region is among the sites in which levels of torsinA transcript [82–85] and protein [86] are highest. Also the predominance of pyramidal neurons with tall somata in our CA3-CA1 hippocampal cultures enables imaging of the neuronal signals with minimal stray signal from the glial cells [60] that typically underlie neuronal monolayer in culture [87].

For staining, we used the fluorescent ceramide analogue BODIPY FL C_5_-ceramide. The dye is thought to be taken up into the ER and transported to the Golgi apparatus based on an active, temperature-sensitive process [47, 88, 89], and it accumulates to a high concentration therein. In the Golgi apparatus, the dye is concentrated, forms excimers and emits in the red range when excited by light in the blue range [47, 48]. In contrast, when located diffusely and non-specifically in the ER, mitochondria and nuclear envelope, the dye emits in the green range due to low concentration [47, 48]. We confirmed that in our cultured neurons, the specificity of Golgi identification was higher when a red emission filter (arrow, Fig 1A; S1A Fig) vs. a green band-pass or green long-pass emission filters was used, although some background staining was visible in the ER. Hereafter we imaged the dye only in the red emission range.

Counterstaining of the nuclei with Hoechst dye showed that the major Golgi staining was paranuclear (Fig 1B; S1B Fig), as reported previously [1, 7]. The Golgi apparatus was indistinguishable qualitatively in hippocampal neurons cultured from WT, HET and HOM mice (Fig 1C).

### Setting intensity threshold for quantitative analysis of the stained Golgi apparatus

In order to compare potential changes in the staining intensity and size of the Golgi apparatus among different genotypes, we carried out quantitative analysis of the images. This required setting two parameters, the first of which was the intensity threshold for positive staining.

**Fig 2:**
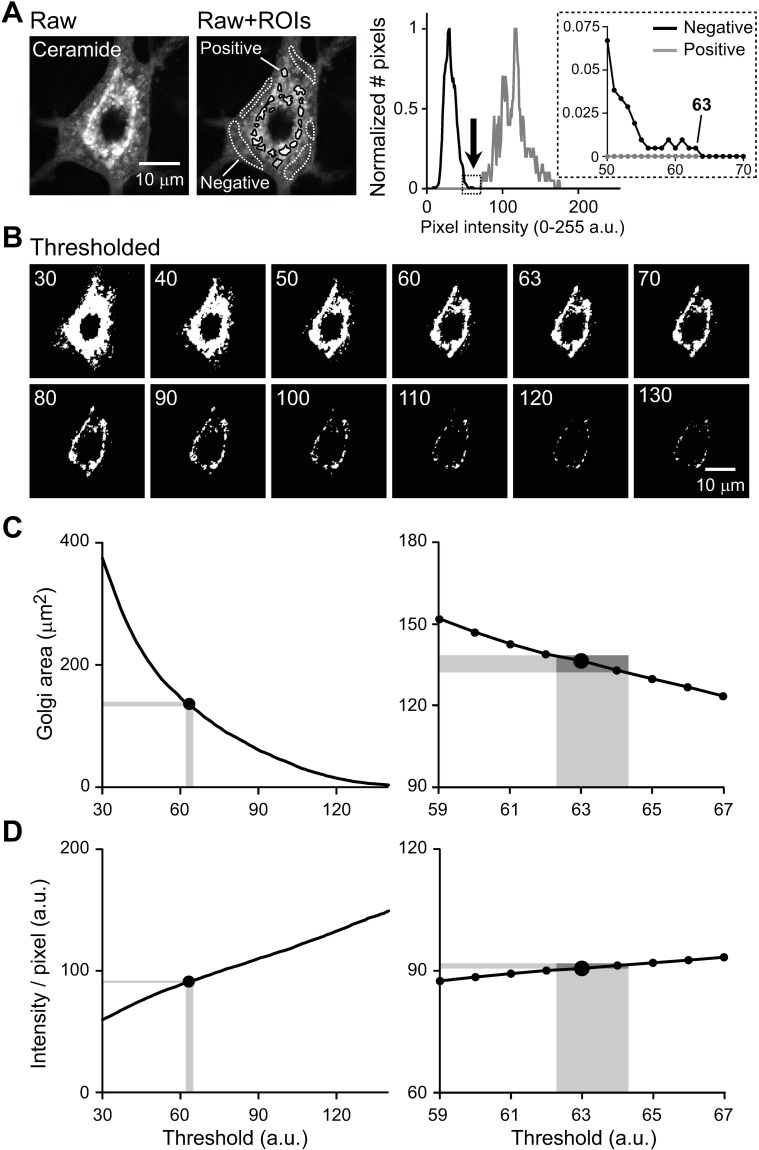
Setting the intensity threshold for quantitative analysis of the ceramide-stained Golgi apparatus. A single image was obtained as part of a z-stack of images by confocal fluorescence microscopy, from a cultured WT hippocampal neuron at 17 DIV. **A:** Method for setting the intensity threshold. An image of a single neuron is shown without (left, Raw) and with (middle, Raw+ROIs) manually assigned regions of interest (ROIs). The ROIs were selected within areas representing cytoplasmic background (Negative), as well as within areas with positive staining (Positive). Histogram (right) shows the distribution of the pixel intensity of the negative, background (black) and positive (gray) staining. The arrow points to the maximal pixel intensity of the background, which was used as the threshold value. In each distribution, the Y-axes of the histograms are normalized to the peak incidence. The inset magnifies the boxed part of the main histogram. **B:** Effects of different threshold values on the area covered by pixels whose intensities are above the threshold. Each panel represents the same image but thresholded at the value indicated. The images are shown in binary format to more clearly illustrate the changes in area (Thresholded). **C:** Dependence of the measured Golgi area on the threshold values for the same image (left). A magnified section of the graph is also shown (right). **D:** Plot similar to that in panel C, but showing the dependence of average pixel intensity on threshold values. In **B-D**, the images and curves represent the data when the threshold was forced to take the indicated values. In **C,D**, vertical and horizontal gray zones show the standard deviations (SDs) of the thresholds, and the corresponding areas and average pixel intensities, when the threshold value was measured repeatedly using the method illustrated in panel **A** (maximal intensity of background).

The intensity threshold was determined individually for each image, as the maximal intensity of the background staining in that neuron (Fig 2A). For this purpose, we set ROIs manually in the areas of somatic cytoplasm in which signal was diffuse and uniform, and could thus be considered background (ER region, "Raw + ROIs"). The maximal intensity of this negative staining (arrow in histogram, "Negative" black trace) was used as the intensity threshold for that image. For comparison, an intensity histogram of brighter regions was overlaid (corresponding to Golgi signal, "Positive" gray trace). Note that the latter is a fraction of the Golgi signal because ROIs were chosen manually for illustrative purpose.

We tested the effects of different threshold values on our measurements of Golgi area and intensity. When the threshold value was varied, there was a corresponding change in the area covered by the pixels whose intensity was above the threshold (Fig 2B). The area (Fig 2C) and average pixel intensity (Fig 2D) were dependent on the threshold values. However, the current method for measuring the threshold values (Fig 2A) yielded consistent results, with small standard deviations across multiple measurements of the same image (gray zones in Fig 2C and 2D). The coefficient of variation (CV = standard deviation / mean) of the measured threshold was 1.6% (mean = 63.4, standard deviation = 1.0, n = 10 measurements of the same image). CVs of the measured area and intensity were 2.4% and 0.7%, respectively (mean = 134.8, standard deviation = 3.2; mean = 91.0, standard deviation = 0.8). Thus we expect that the measurement error will be on the order of 1–3%.

A number of other intensity thresholds were tested, including the numerical value above the mean intensity of the cytoplasmic background (e.g. 3 times the standard deviation above the mean), the minimal intensity value of the strongly stained area (Golgi), the value at which the histograms of the cytoplasmic background and Golgi intersected (when there is such an intersection), or the value at which the overall soma, including Golgi and cytoplasmic background, demonstrates a notch in the intensity histogram. Among these, the method described in the previous paragraphs was found to be most reliable and most objective. It was least affected by different areas and intensities of the Golgi apparatus across neurons.

### Selecting optical sections for quantitative analysis of the stained Golgi apparatus by the 3-image average method

**Fig 3:**
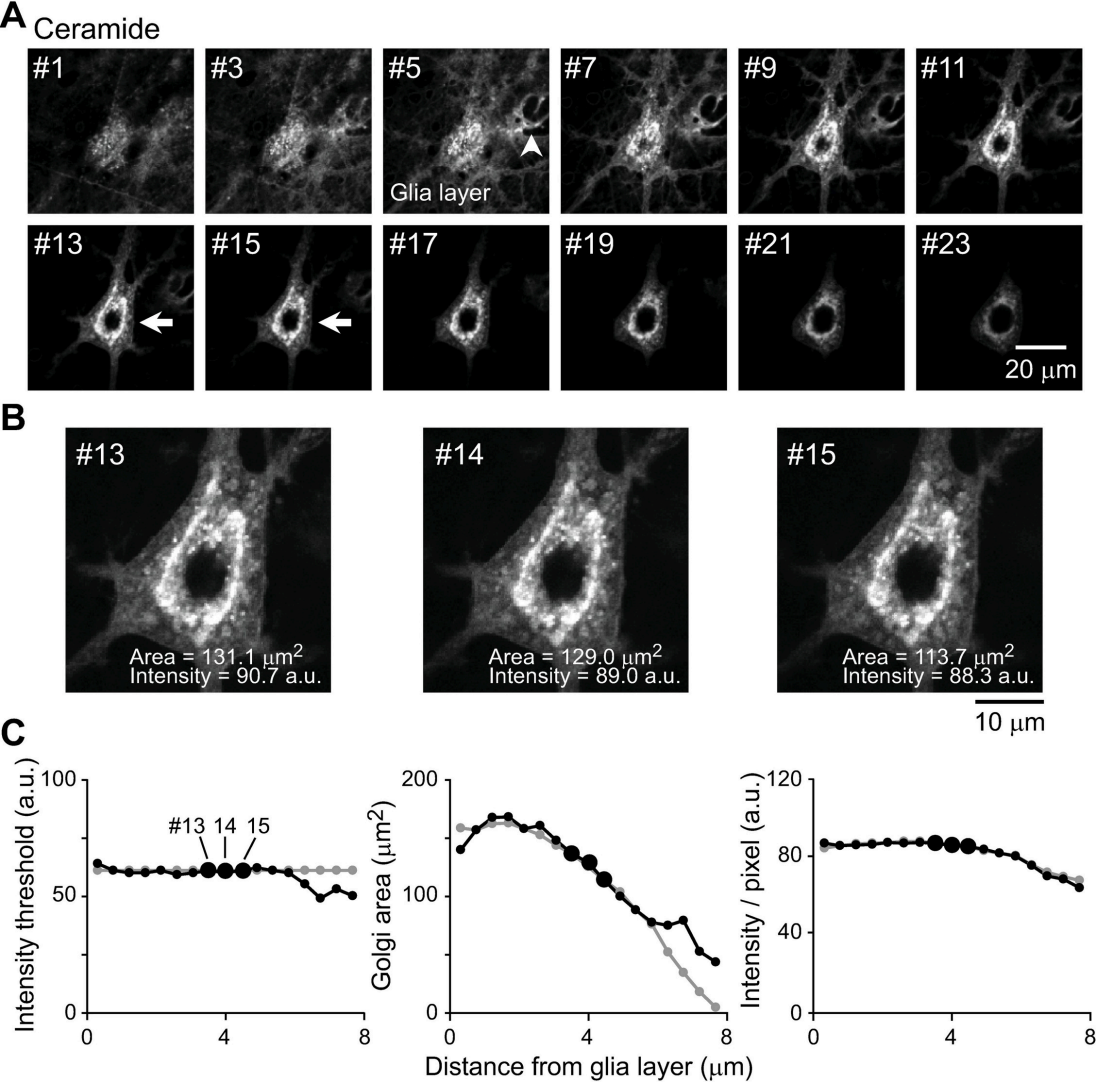
Selecting images in a z-stack for quantitative analysis of the ceramide-stained Golgi apparatus. All images were obtained by confocal microscopy. **A:** Multiple images in a z-stack of the neuron imaged in Fig 2. Arrowhead indicates the level of maximal glial signal. Arrows indicate the optimal levels of neuronal signal. Individual images in a z-stack were acquired at 0.44-μm intervals; distance between the panels is 0.44 *×* 2 = 0.88 μm. **B:** Magnified views of images #13–15 in A. The measured Golgi areas and intensities are indicated. **C:** Measured intensity threshold, Golgi area and average pixel intensity of the images shown in panel A. These values depend on the distance from bottom of glial layer. Small black dots indicate the values measured when the intensity threshold was determined for each image. Large black dots indicate the values for images #13–15, which were ultimately used to report Golgi parameters. For comparison, the gray dots indicate the values measured when the intensity threshold was fixed at 63, the value determined for image #14.

**Fig 4:**
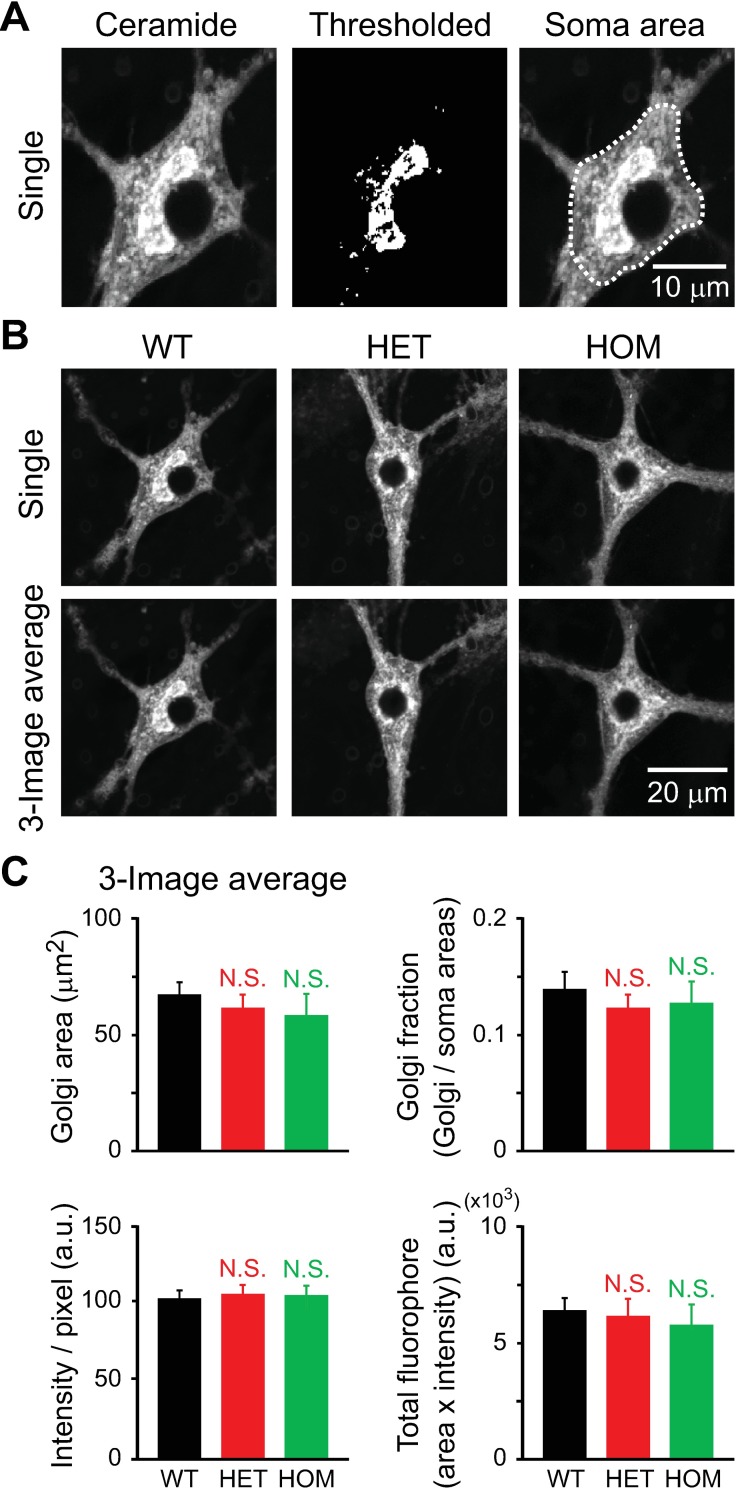
Structure of the Golgi apparatus in hippocampal neurons of ΔE-torsinA knock-in mice, as analyzed using the 3-image average method. Confocal fluorescence microscopy of cultured neurons at 17–19 DIV. **A:** Representative confocal image of a WT neuron stained with BODIPY FL C_5_-ceramide (left). Thresholded image is shown in binary format (middle). The ROI used to measure the somatic area is overlaid on the image (right). **B:** Representative confocal images of neurons of the WT, HET and HOM genotypes. An optical section is shown (Single), as a comparison for an image averaged from 3 sections used for analyses. **C:** Quantitative analysis of measured parameters. Total fluorophore (BODIPY FL C_5_-ceramide) was calculated as (area) *×* (averaged intensity / pixel). Columns represent the means and the bars represent standard errors of the mean (SEM). There was no statistical significance (NS) in the values measured for mutants vs. WT (p>0.1; *t*-test; n = 20, 26, 18 neurons for WT, HET and HOM, respectively).

The second requirement for quantitative analysis was selecting optical sections from a given neuron for assessment. We tested two methods. In one (Figs 3 and 4), we averaged measurements from 3 consecutive images in a z-stack.

Images of Golgi revealed variability even within a single z-stack series. Glial cells reside on the coverslip and can contribute to the signal. Indeed, near the bottom of a z-stack, glial staining contributed to the signal (images #1–9 in Fig 3A) with maximum intensity achieved near image #5 (arrowhead). The true level of coverslip surface was unclear, because the large point-spread function in the axial direction makes it possible for signal from the smallest resolvable object (<250 nm in diameter) to spread >1 μm along the z-axis [90]. Neuronal staining was detected in all images (#1–23, Fig 3A). In images #13–15, the dendrites were clearly visible, and the neuronal Golgi appeared to be independent of the glial signal, large in size, and intensely stained (arrows, Fig 3A and 3B). In subsequent images (images #>15), the Golgi size was smaller and the signal intensity lower, partly due to biology of the cell, but also the spherical aberration and refractive index mismatch in the specimen [90, 91].

When the intensity threshold was set in each image of the z-stack as described above (Fig 2A), there were small variations in the measured area (number of pixels) and intensity of the pixels whose values were above the threshold (small black dots in Fig 3C). In contrast, when the threshold value was fixed at a single value (63) for all images, the measurements differed significantly, especially where the images were far from the coverslip (gray dots in Fig 3C).

In order to minimize variation, we measured and averaged the areas and pixel intensities in 3 consecutive images near the level of the dendrites, based on the intensity threshold determined for each image (large black dots in Fig 3C, 3-image average method). The advantages of this method are that: 1) glial signal is excluded, 2) the neuronal Golgi apparatus is included at its broadest level, 3) the dendritic outposts are included when present, and 4) the impact of optical artifacts far from the coverslip is minimized.

### Quantitative analysis of area and intensity of the stained Golgi apparatus

Based on the above-described methods for setting intensity threshold and selecting images to analyze, we assessed the structure of the Golgi apparatus in the cultured hippocampal neurons of WT, HET and HOM mice (Fig 4). Additionally, we measured the somatic area including the nucleoplasm (Fig 4A). There was no genotypic difference in the measurements of Golgi area, the fraction of the soma that was occupied by the Golgi apparatus (Golgi area / somatic area), the average intensity of pixels, or the total amount of fluorophore (Golgi area *×* average pixel intensity) (p>0.1; not significant (NS) with respect to WT values; n = 18–26 neurons per genotype) (Fig 4B and 4C). The area is representative of the overall size of the Golgi apparatus, and the intensity reflects multiple factors, including the surface area of the Golgi cisternae at the ultrastructural level, the density of the dye in the Golgi, and mechanisms whereby the dye is transported to the Golgi. We did not observe dramatic changes in morphology, such as Golgi fragmentation, in any genotype. These results indicate that the *Tor1a*^ΔE^ mutation did not affect the Golgi structure or associated factors in cultured hippocampal neurons.

### Quantitative analysis, based on a second method of selecting the optical sections

We validated our results using a second method to select images. Instead of selecting three images in a z-stack, we projected images in a z-stack onto a single plane, using the maximum intensity projection (MIP) algorithm [76]. The advantage of this method is that it represents signal in all images of a z-stack, and therefore it can minimize the impact of signal variability in a small number of selected images. However, it has the disadvantage that it emphasizes signals from glia. Given that these are closer to the coverslip, they undergo less depth-dependent signal decay than neurons which lie farther from the coverslip. We circumvented this disadvantage by excluding images that had glial contamination (as in the 3-image averaging method, Fig 3).

**Fig 5:**
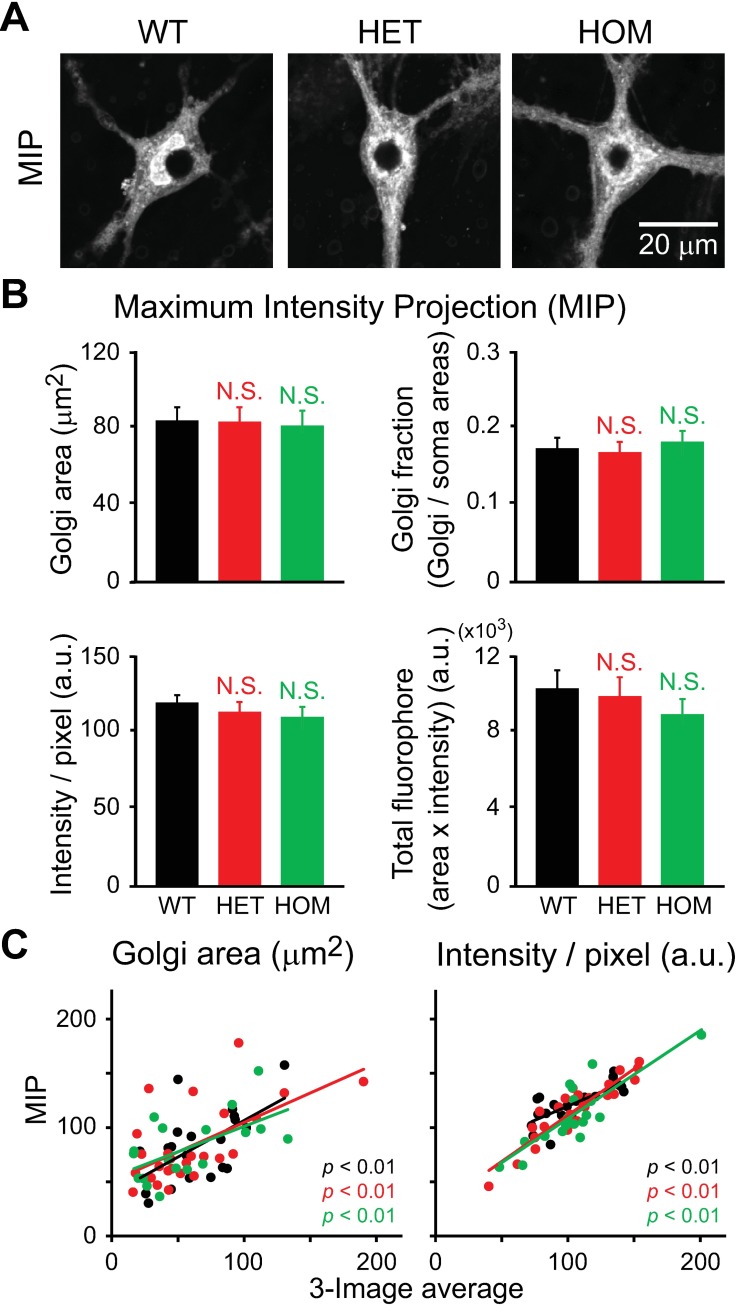
Structure of the Golgi apparatus in the hippocampal neurons of ΔE-torsinA knock-in mice, as analyzed using the maximum intensity projection (MIP) method. Confocal fluorescence microscopy of cultured neurons at 17–19 DIV. **A:** Representative images of the WT, HET and HOM neurons shown in Fig 4B, stained with BODIPY FL C_5_-ceramide and but shown after MIP. **B:** Quantitative analysis of indicated parameters. Columns represent the mean and the bars represent the SEM. Differences in values measured for mutant vs. WT neurons were not statistically significant (p>0.1; *t*-test; n = 20, 26, 18 neurons for WT, HET and HOM, respectively). **C:** Positive correlation between results obtained using the 3-image averaging and MIP methods (p<0.01; *t*-test for Pearson correlation coefficient; same number of neurons as in panel B).

We applied the MIP method to the same set of data (Fig 5A), and found that there was no genotypic difference in the four parameters described above (p>0.1; n = 18–26 neurons per genotype; Fig 5B). Further analysis showed positive correlations between the parameters, as measured using the 3-image averaging and MIP methods (p<0.01, for the three genotypes) (Fig 5C). This indicates that MIP can be used in place of the more labor-intensive, 3-image averaging method. The degree of correlation was lower for area (left) than for intensity (right). In some neurons, the measured area was greater when MIP vs. the 3-image averaging method was used. This was because, when a part of the Golgi apparatus was positioned above or below nucleus, MIP included the Golgi signals in the projected nuclear region, whereas 3-image averaging effectively excluded such signals.

### Quantitative analysis of Golgi apparatus of neurons from the cerebral cortex and striatum

In order to extend the quantitative analyses to other neurons, the maximum intensity projection method was applied to the cultured neurons of the cerebral motor cortex and striatum.

The cerebral cortex contributes to the defects in motor control that are associated with dystonia [92], and conditional knock-out of torsinA in this brain region induces motor abnormalities in mice [93]. The striatum is also relevant to motor control [22, 92, 94]. Striatum-specific conditional knock-out of torsinA results in motor deficits [95], and the striatal neurons show subtle structural changes in ΔE-torsinA knock-in mice [96]. In addition, glutamatergic transmission at the cortico-striatal synapse is enhanced when ΔE-torsinA is overexpressed pan-neuronally in transgenic mice [64, 97, 98] and rats [99], or when ΔE-torsinA is expressed at endogenous levels in ΔE-torsinA knock-in mice [100, 101].

**Fig 6:**
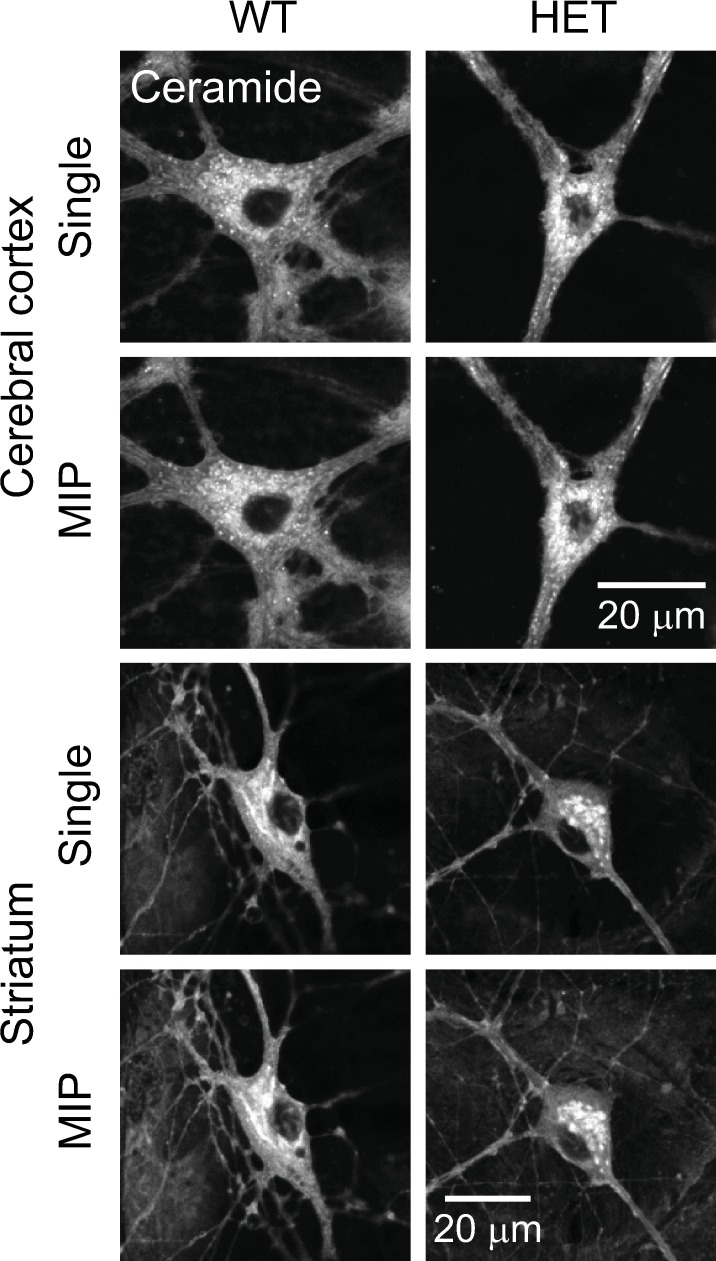
Structure of the Golgi apparatus in neurons from the cerebral cortex and striatum of WT and ΔE-torsinA knock-in mice. Confocal microscopy of WT and HET neurons stained with BODIPY FL C_5_-ceramide, at 17–19 DIV. Single and MIP images are shown.

**Fig 7:**
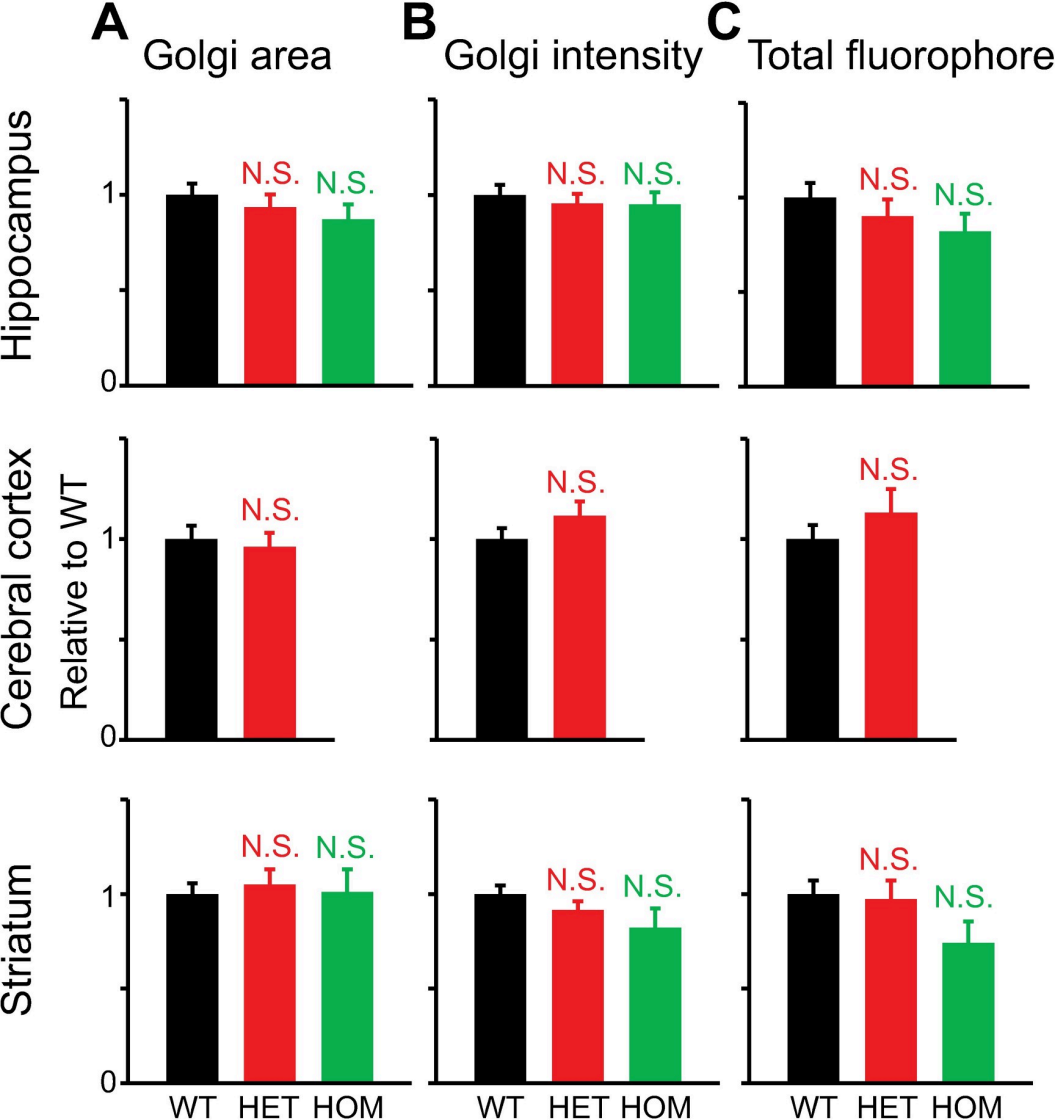
Quantitative analysis of the Golgi structure in ceramide-stained neurons from the hippocampus, cerebral cortex and striatum, as analyzed by the MIP method. Confocal microscopy was carried out at 17–21 DIV. **A:** Golgi area. **B:** Averaged pixel intensity in the Golgi. **C:** Total amount of fluorophore. All measured values were normalized to average values of WT neurons. Differences in values measured for mutant vs. WT neurons were not statistically significant (p>0.1; *t*-test; n = 31, 40, 20 hippocampal neurons, 47, 40 cerebral cortical neurons, and 40, 45, 30 striatal neurons for WT, HET and HOM, respectively).

BODIPY FL C_5_-ceramide was used to stain the Golgi apparatus in live neurons obtained from the cerebral cortex and striatum of WT and HET mice (Fig 6). We did not observe noticeable changes, such as Golgi fragmentation, in either genotype. Consistent with our findings in hippocampal neurons, the Golgi area (Fig 7A), the pixel intensity in the Golgi (Fig 7B) and the total amount of fluorophore (Fig 7C) in the HET neurons were indistinguishable from those in WT neurons in these brain regions (p>0.1; n = 20–47 neurons per genotype per brain region). Thus the *Tor1a*^ΔE^ mutation did not affect the Golgi structure in cultured neurons of the cerebral cortex or striatum.

### Extent to which the Golgi apparatus encircles the nucleus

**Fig 8:**
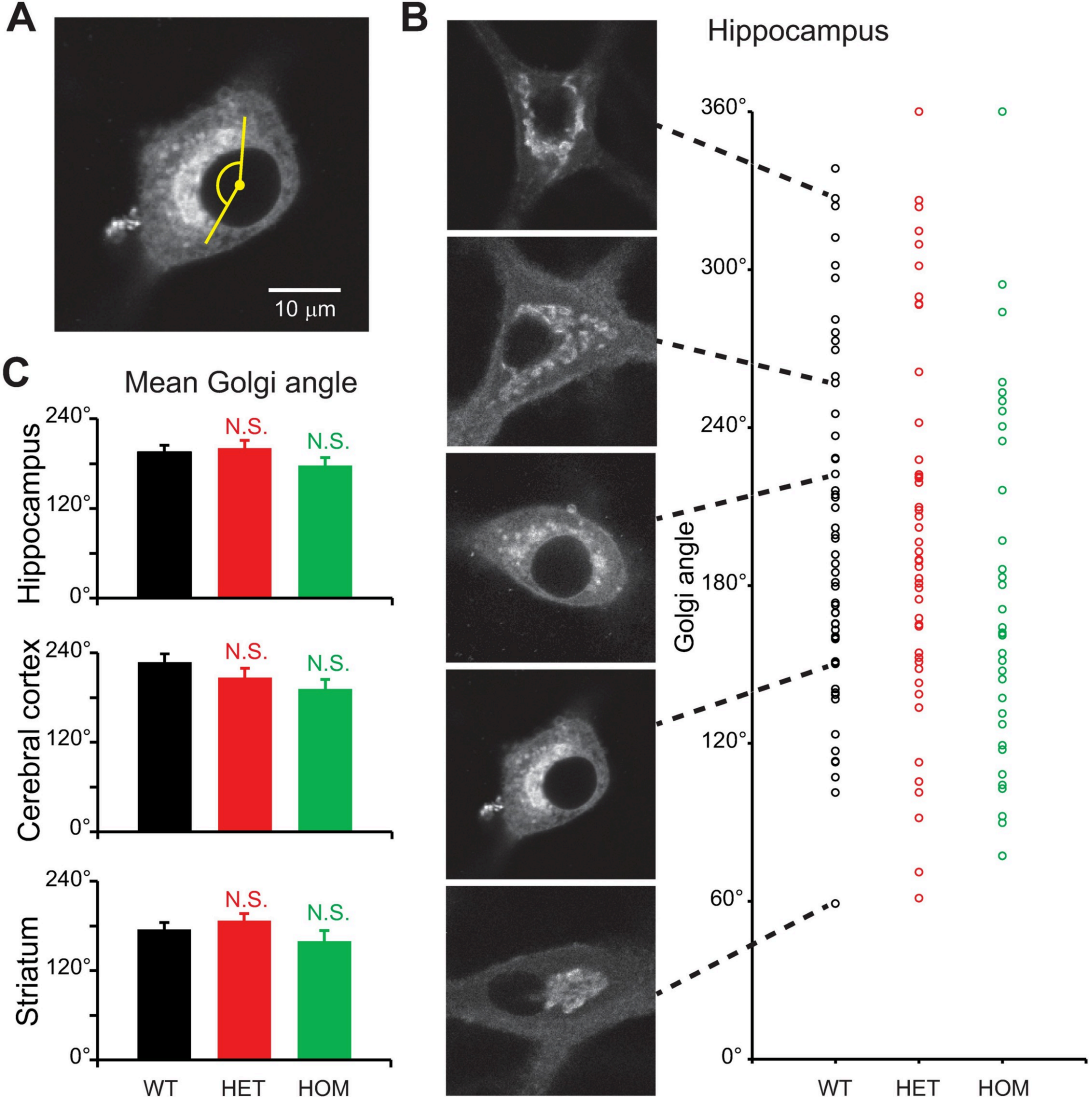
Degree to which the Golgi apparatus encircled nuclei. Confocal microscopy of neurons stained with BODIPY FL C_5_-ceramide, carried out at 17–19 DIV. **A:** Representative confocal image of a WT hippocampal neuron. The extent to which the Golgi apparatus encircled the nucleus was measured as the angle subtended by the Golgi, with the center of the nucleus defined as the vertex. **B:** Variability of angle subtended by the Golgi in hippocampal neurons. Each circle represents a single neuron. **C:** Differences in values measured for mutant vs. WT neurons were not significantly different (p>0.1; *t*-test; n = 54, 49, 37 hippocampal neurons, 49, 40, 25 cerebral cortical neurons, and 53, 58, 29 striatal neurons for WT, HET and HOM, respectively).

We also evaluated the extent to which the Golgi apparatus encircled the nucleus. For this purpose, we measured the angle subtended by the Golgi, as seen from the center of nucleus (Fig 8A). We found this feature of Golgi shape to be very heterogeneous, even among neurons of a given genotype and in the same brain region (Fig 8B). However, there was no genotype-dependent statistical difference in this parameter (p>0.1; n = 25–58 neurons per genotype per brain region; Fig 8C).

### Fixed-cell staining of the Golgi apparatus

**Fig 9:**
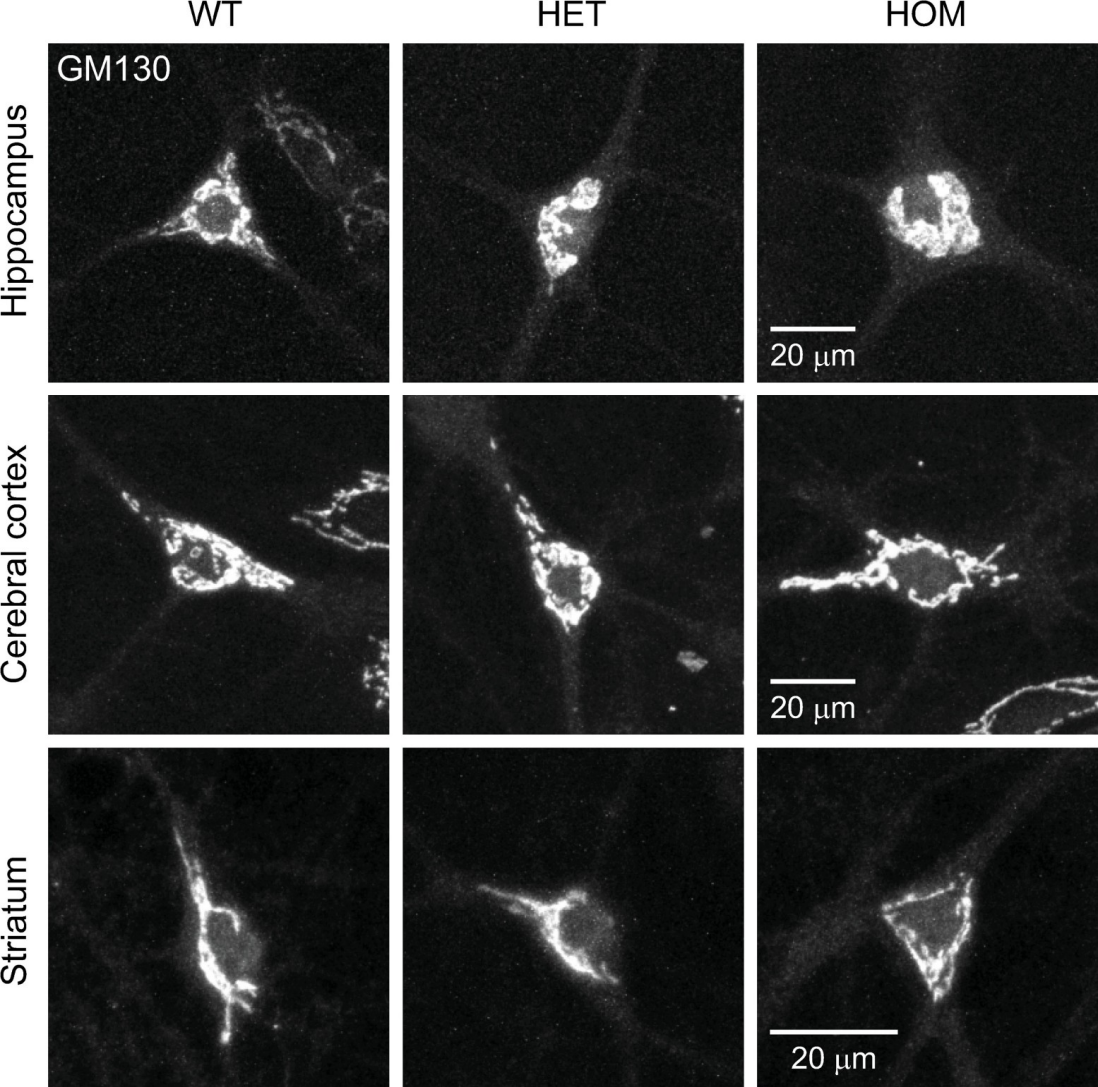
Structure of the Golgi apparatus, as visualized by GM130 immunocytochemistry. Representative confocal images of cultured hippocampal, cerebral cortical and striatal neurons, obtained from WT, HET and HOM mice. MIP images are shown, and were acquired at 17–19 DIV.

**Fig 10:**
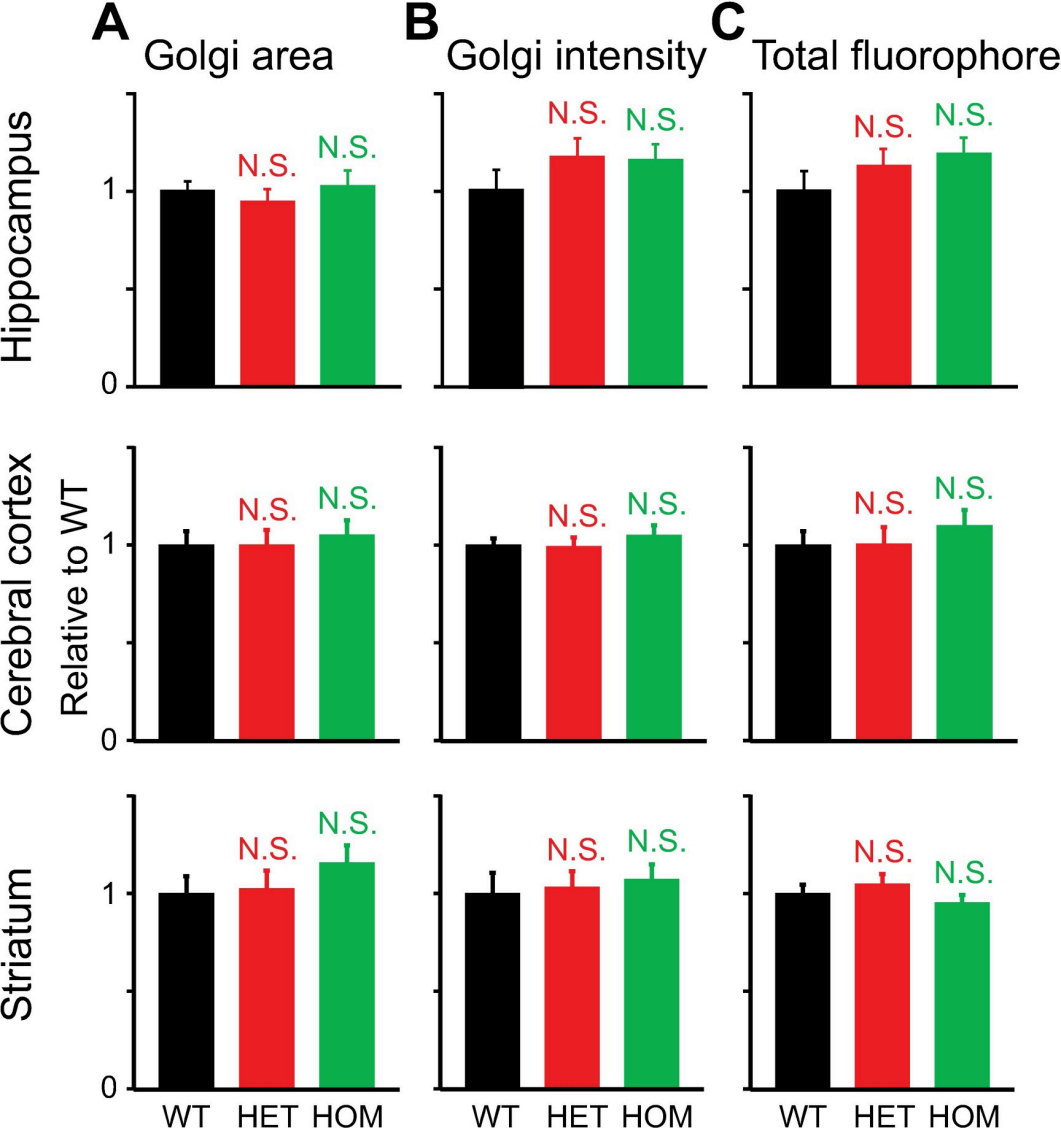
Quantitative analysis of Golgi structure as visualized by GM130 immunocytochemistry. The stained structure was analyzed using the MIP images. Confocal microscopy was carried out at 15–21 DIV. **A:** Golgi area. **B:** Intensity / pixel in the Golgi. **C.** Total fluorophore in the Golgi. All measured values were normalized to the average value for WT neurons. Differences in values measured for parameters in mutant vs. WT neurons were not statistically significant (p>0.1; *t*-test; n = 12, 18, 13 hippocampal neurons, 20, 19, 20 cerebral cortical neurons, and 19, 18, 19 striatal neurons for WT, HET and HOM, respectively).

The results obtained with the live-cell staining were confirmed by fixed-cell immunocytochemistry. We examined the expression of an endogenous Golgi matrix protein GM130 [51] in neurons using the MIP method. There were no obvious structural differences in the Golgi apparatus in the neurons, regardless of whether they were obtained from the hippocampus, cerebral cortex or striatum, or from WT, HET or HOM ΔE-torsinA knock-in mice (Fig 9). There was also no genotypic difference in the Golgi area (Fig 10A), pixel intensity (Fig 10B) or total fluorophore (Fig 10C) in any brain region (p>0.1; n = 12–20 neurons per genotype per brain region).

### Changes during culture period

**Fig 11:**
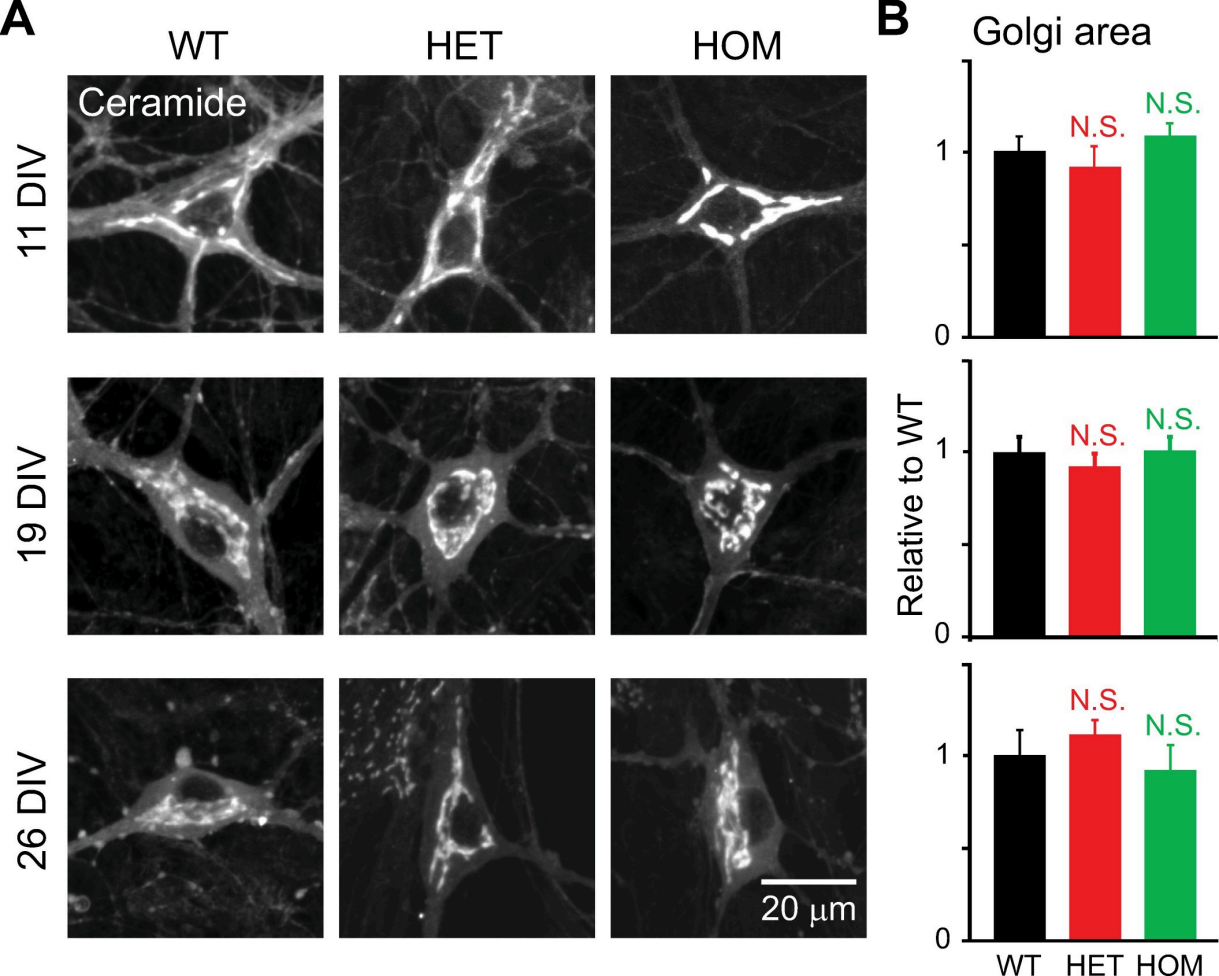
Structure of the Golgi apparatus during the course of culture. Hippocampal neurons of ΔE-torsinA knock-in mice were stained with BODIPY FL C_5_-ceramide, and imaged by confocal microscopy at the specified DIV. **A:** Representative confocal images of neurons obtained from WT, HET and HOM ΔE-torsinA knock-in mice, at 11, 19 or 26 DIV. MIP images are shown. **B:** Differences in Golgi area between mutant and WT neurons, as analyzed by the MIP method, were not statistically significant during maturation (p>0.1; *t*-test; n = 26, 14, 18 11-DIV neurons, 30, 44, 32 19-DIV neurons, and 11, 17, 18 26-DIV neurons for WT, HET and HOM, respectively). All values measured were normalized to the average WT value.

Having analyzed neurons at 17–21 DIV, we next evaluated changes in the Golgi over the course of neuronal culture. They were analyzed at 11, 19 and 26 DIV (Fig 11A). Staining with BODIPY FL C_5_-ceramide did not reveal drastic changes in the structure of cultured hippocampal neurons, such as fragmentation, in any genotype at any stage of maturation. Moreover, the overall area of the Golgi apparatus, as measured by MIP, did not differ by genotype (p>0.1; n = 11–44 neurons per genotype; Fig 11B).

**Fig 12:**
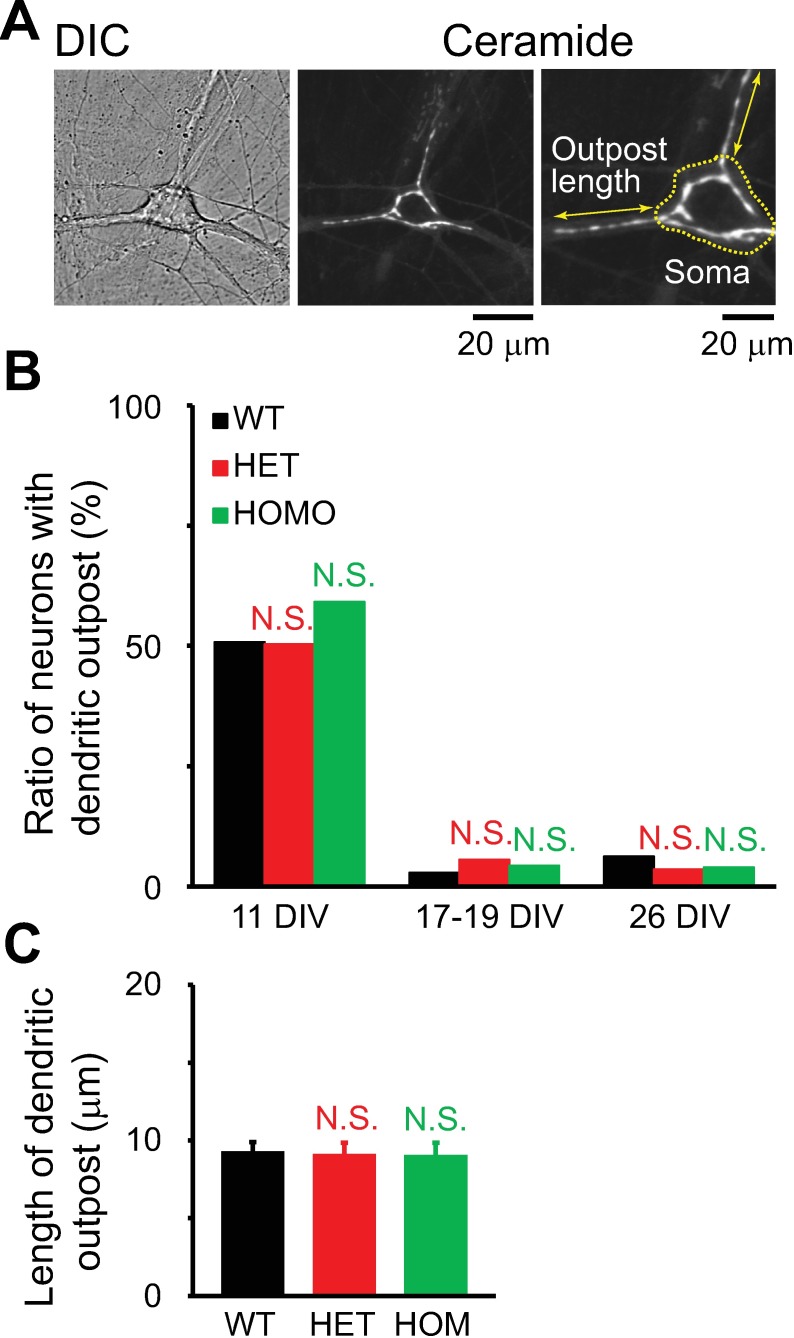
Dendritic Golgi outposts in young cultures. **A:** DIC and confocal images of a cultured, BODIPY FL C_5_-ceramide-stained hippocampal neuron at 11 DIV. Double-ended arrow indicates the length of longest outpost in this neuron. **B:** Ratios of neurons with outposts to total number of neurons. The ratios between mutant and WT neurons were not statistically significant (p>0.1; chi-squared test; total number of neurons = n = 191, 143, 98 11-DIV neurons, 35, 54, 46 17-to-19-DIV neurons, and 16, 28, 25 26-DIV neurons for WT, HET and HOM, respectively). **C.** Lengths of dendritic outposts. Differences in lengths of dendritic outposts between mutant and WT neurons were not statistically significant (p>0.1; *t*-test; n = 97, 72, 58 neurons for WT, HET and HOM, respectively). In **B** and **C**, the data obtained with ceramide staining and immunocytochemistry for GM130 were merged.

Since extensions of the Golgi into the dendrites (dendritic outposts) control dendritic growth [11] and have been reported to change over the course of maturation in hippocampal cultures [76], we evaluated them in our hippocampal culture system. At 11 DIV, the outposts were present in thick dendrites of some neurons, and occasionally in multiple dendrites of a single neuron (Fig 12A). At 11 DIV, ~50% of neurons of all genotypes had outposts. This number was significantly reduced in older neurons (Fig 12B), but there was no genotype-associated difference at any age in culture (p>0.1; n = 16–191 neurons per genotype). At 11 DIV, when many outposts were present, there was no genotype-specific difference in the length of the longest outpost of a neuron (p>0.1; n = 58–97 neurons per genotype; Fig 12C). Thus, the *Tor1a*^ΔE^ mutation did not affect Golgi structure during culture period.

### Degree of polarization

**Fig 13:**
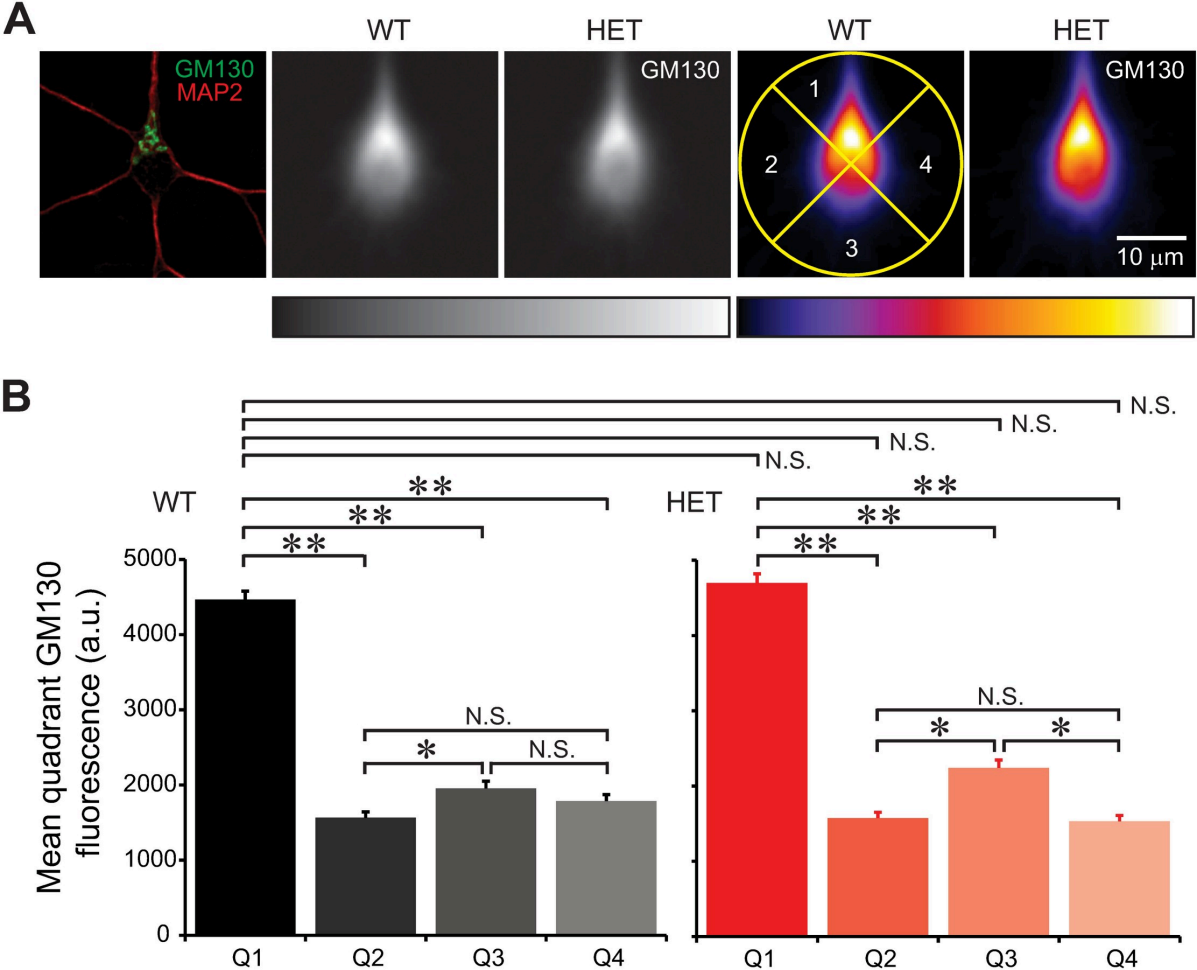
Degree of Golgi polarization at the base of thickest dendrite. Hippocampal neurons were stained for GM130 at 11 DIV, centered, and oriented such that the thickest dendrite pointed toward 12 o'clock, and averaged. The polarity of the Golgi was analyzed as the distribution of GM130 intensity in the top quadrant (90° pie) relative to the other quadrants. **A:** The leftmost panel shows the image of a single neuron, stained by immunocytochemistry for GM130 and a dendritic marker MAP2 (single confocal plane). Two grayscale images show averaged GM130 signals in neurons from WT and HET neurons (slide scanner images). Two images on the right show the same GM130 signals shown in pseudocolor. The center of each panel corresponds to the somatic center. Four quadrants are illustrated with a yellow circle in one panel. The scale bar applies to all panels. Rectangles below the images show the corresponding color lookup tables. **B:** Relative distributions of the Golgi in the four quadrants. Golgi was polarized in the top quadrant (Quadrant 1, Q1) in WT and HET neurons (**: p<1.01 *×* 10^−46^; *: p<1.77 *×* 10^−3^). However, differences in polarization between mutant and WT neurons were not statistically significant (N.S.; p>2.61 *×* 10^−2^ with α = 1.78 *×* 10^−3^ after Bonferroni correction; *t*-test; n = 410 and 407 neurons for WT and HET, respectively).

In the soma of hippocampal and cerebral cortical neurons, the Golgi apparatus is oriented toward the longest (thickest) dendrite, and this Golgi polarity is associated with growth of that dendrite [11]. We used the same approach to test whether this polarity is maintained in HET neurons. Images of GM130 staining were centered on the somatic center (identified in MAP2 staining) and oriented with respect to the thickest dendrite. The signal was then averaged (Fig 13A) and the relative distribution of the Golgi in each quadrant (90° pie) analyzed (Fig 13B). In both WT and HET hippocampal neurons, the Golgi was most abundant in the top quadrant, in comparison with other quadrants. This indicates that the Golgi was polarized toward the base of the thickest dendrite. However, there was no genotype-specific difference between the same quadrants, suggesting that the *Tor1a*^ΔE^ mutation did not affect the Golgi distribution (n = 407–410 neurons per genotype).

### Western blotting of GM130 in neuronal cultures

**Fig 14:**
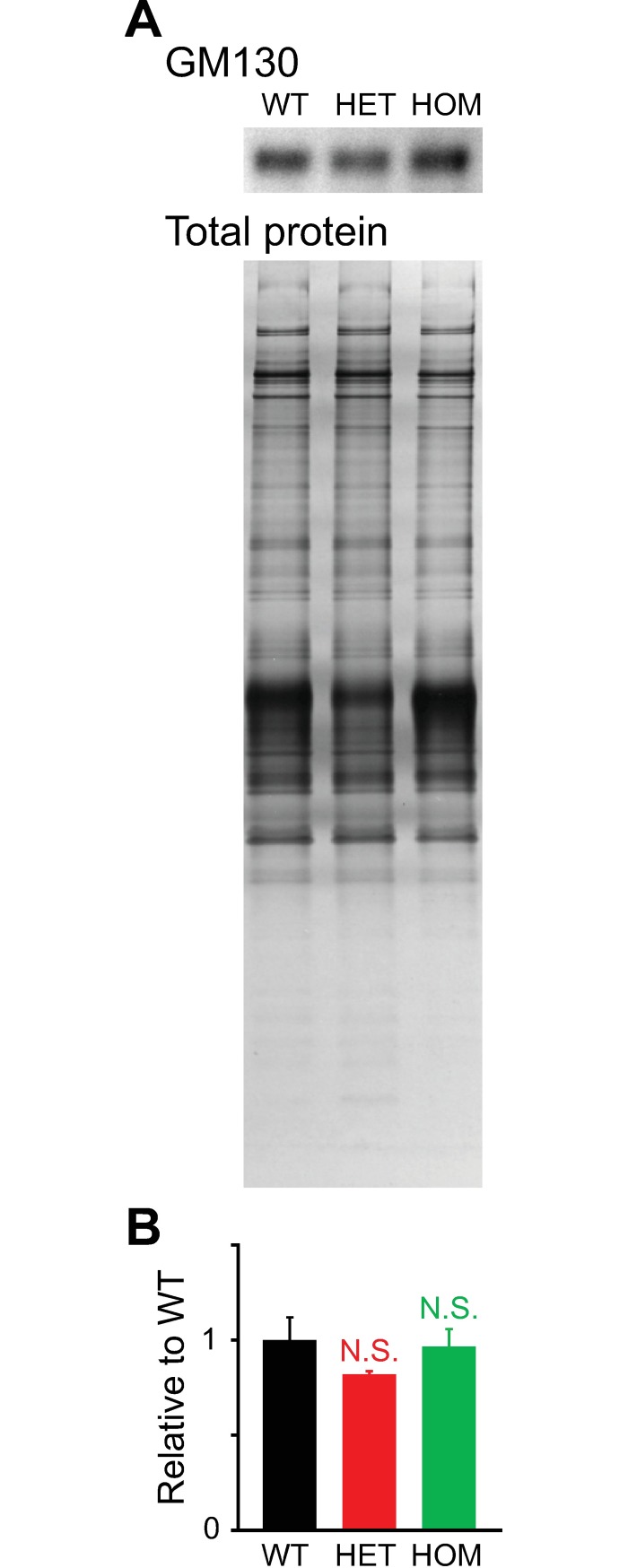
Expression of the Golgi protein GM130 in cultured cells as assessed by Western blotting. Cultured hippocampal cells were processed for Western blotting at 17 DIV. **A:** GM130 immunoblot, with total protein used as a loading control. **B:** Bar graph of GM130 levels following normalization to the average WT value. Differences in expression between mutant vs. WT cultures were not statistically significant (p>0.1; *t*-test; n = 3 per genotype).

We used Western blotting to assess GM130 expression in the hippocampal cultures (Fig 14). As a loading control, we used total protein [71, 73] with the amount measured by silver staining [72]. We used this method instead of relying on levels of a single protein as a loading control, because the *Tor1a*^ΔE^ mutation affects the translational and transcriptional regulation of commonly used reference proteins such as glyceraldehyde 3-phosphate dehydrogenase (GAPDH) [102, 103], actin and tubulin [103]. TorsinA is also known to interact with some of these proteins (actin and tubulin) [104].

For each genotype, the anti-GM130 antibody stained a band at the expected size (Fig 14A, S2 Fig). The optical density of the GM130 band did not change in association with the genotype (p>0.1 for HET vs. WT and HOM vs. WT; n = 3 per genotype) (Fig 14B).

### Degree of Golgi disruption

**Fig 15:**
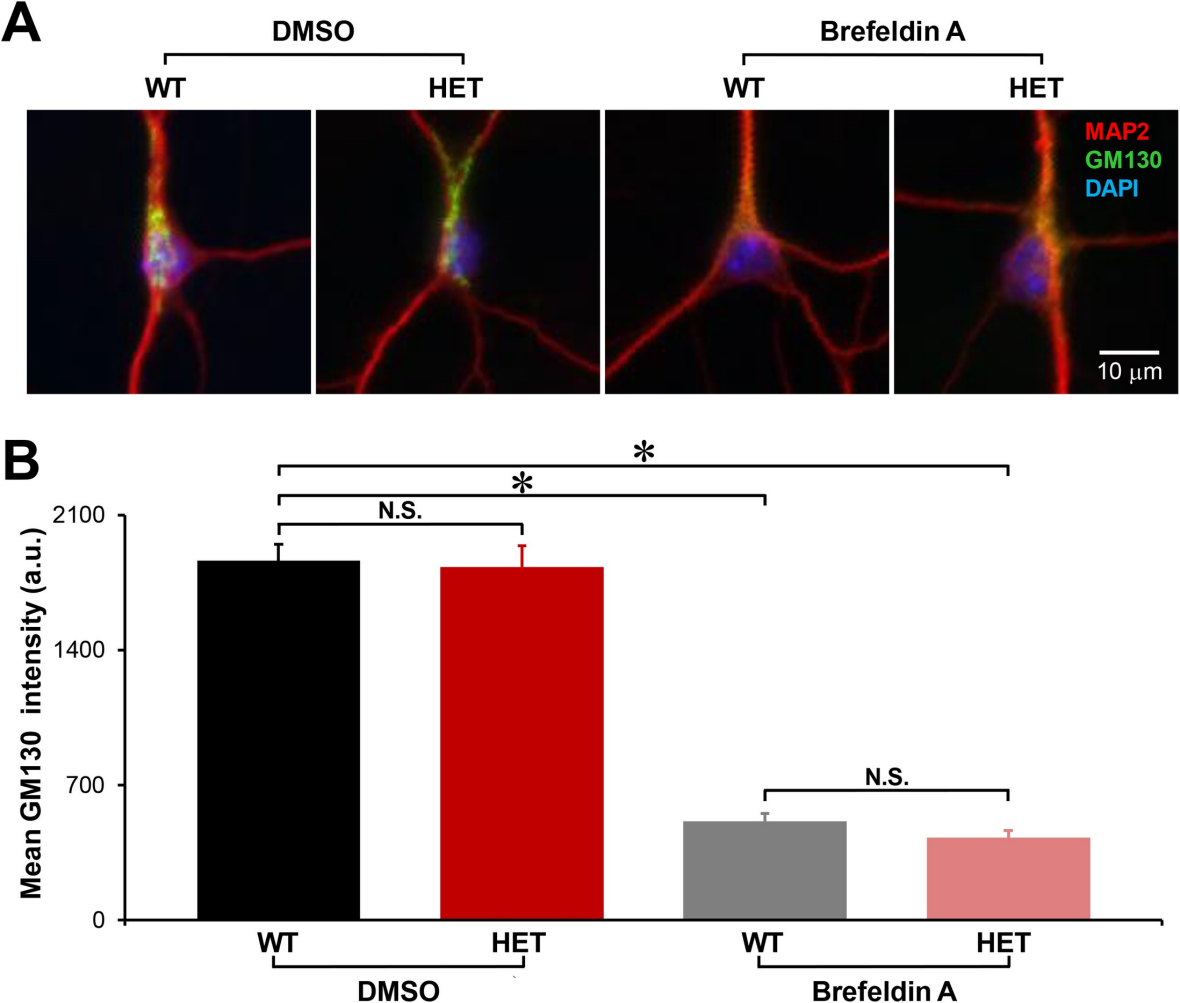
Degree of Golgi apparatus disruption by brefeldin A. Cultured hippocampal neurons at 11 DIV, following treatment with 1 μg/ml brefeldin A or DMSO for 2 hours at 37°C and immunocytochemical staining for GM130, MAP2 and DAPI. **A:** Representative slide scanner images of neurons. The images are shown in an 8-bit intensity format. The same image contrast was used in each color channel (representing GM130, MAP2 or DAPI) throughout the 4 images, such that the minimum of 4 minima and the maximum of 4 maxima were 0 and 255 on an 8-bit intensity scale. The mean intensity values of GM130 in the 4 images were (from left to right): 745, 730, 261 and 281. **B:** Quantitation of GM130 staining intensity. There was no genotypic difference in the GM130 staining intensities when the neurons were treated with vehicle (N.S.; p = 0.822). In both genotypes, treatment with brefeldin A reduced the GM130 staining intensity in comparison to treatment with vehicle (WT, *: p = 3.04 *×* 10^−32^; HET, *: p = 3.52 *×* 10^−18^). However, there was no genotypic difference in the GM130 staining intensities after treatment with brefeldin A (N.S.; p = 0.139) (n = 139 WT and 62 HET neurons treated with DMSO only, and 85 WT and 70 HET neurons treated with brefeldin A).

Finally, we tested to what extent the HET neurons respond to a Golgi-disrupting agent. Both WT and HET hippocampal neurons were treated with 1 μg/ml brefeldin A or vehicle (DMSO) for 2 hours at 37°C. Brefeldin A has been reported to cause the Golgi apparatus to fragment and disperse throughout the cytoplasm in 15–60 min [105–107]. We prolonged the treatment to 2 hours to ensure the maximal effects. As expected. the treatment with brefeldin A disrupted the Golgi apparatus (Fig 15A), whereas treatment with vehicle did not. In addition, the latter did not lead to significant differences in GM130-staining between WT and HET neurons (p>0.8; n = 139 and 62 neurons, respectively; Fig 15B), confirming the previous results without such treatments (Figs 4, 5, 7, 10 and 11). Treatment with brefeldin A significantly reduced the average intensities of GM130 in both WT and HET neurons (comparison was to their respective vehicle controls; p<1 *×* 10^−17^; n = brefeldin A-treated 85 WT and 70 HET neurons). However, there was no significant difference in the effects of brefeldin A on the final GM130-staining intensity in HET vs. WT neurons (p>0.1). These results suggest that the *Tor1a*^ΔE^ mutation did not affect the sensitivity to the Golgi-disrupting agent.

## Discussion

This is the first report on a detailed structural analysis of the Golgi apparatus in a DYT1 dystonia model. Neither live- nor fixed-cell staining of the neuronal Golgi apparatus revealed morphological differences in either HOM or HET vs. WT mice. In the three brain regions examined, the size, staining intensity, shape and localization of the Golgi were indistinguishable across the three genotypes. There were also no signs of general morphological changes such as disintegration, or of defects in the establishment of Golgi outposts over the course of neuronal culture. The staining intensity of Golgi after prolonged treatment with brefeldin A did not differ between mutant and WT neurons, suggesting that the sensitivity to the Golgi-disrupting agent was not affected by the *Tor1a*^ΔE^ mutation.

Changes in the structure of the central nervous system have been reported in HET mice. At the network level, there was thinning of the fiber tracts that link motor circuits, e.g. the ponto-cerebellar, cerebello-thalamic, thalamo-cortical, and thalamo-striatal pathways [108]. Also, at the cellular level, there were fewer dendritic branches and dendritic spines of the striatal medium spiny neurons [96] and the cerebellar Purkinje neurons [109, 110]. At the organelle level, however, no changes have been reported for these mice. Although HOM mice feature an abnormal nuclear envelope that develops membranous vesicle-like structures (“blebs”) at the ultrastructural level, this is not the case for the HET mice [55, 111]. Considering that DYT1 dystonia patients are heterozygous for the mutant allele, this nuclear envelope abnormality in the HOM mice, or at least its morphological aspect, might be only minimally relevant to the dystonia pathophysiology [95].

Our study suggests that the static Golgi morphology might not be a subcellular component of the DYT1 dystonia phenotype. A recent report showed that some of the abnormalities in neuronal function in the HET mouse are limited to certain brain regions (e.g. long-term synaptic plasticity is abnormal at cortico-striatal synapses but normal at hippocampal synapses [101]). However, our finding was consistent across three functionally distinct brain regions (the hippocampus, cerebral cortex, and striatum), and thus might argue against the idea that we have looked in the wrong region.

### Although the size and shape of the Golgi apparatus change under some pathological conditions, they do not in DYT1 dystonia

Dramatic changes in protein levels within the cell can lead to morphological changes in the Golgi apparatus. Several examples are available in the literature. First, genome-wide RNAi screening showed that the most common form of Golgi change associated with targeted knock-down is fragmentation (loss of the paranuclear Golgi signal, with a concomitant increase in the number of signal-positive fragments throughout the cytoplasm) [74]. Less frequently, the Golgi apparatus becomes either "dispersed" (the paranuclear signal remains, but "with a loss in the usual compactness") or "condensed" (the paranuclear signal remains and is more compact).

Second, in the mouse disorder dystonia musculorum, the Golgi apparatus of sensory neurons becomes fragmented. Dystonia musculorum is an inherited recessive sensory neuropathy, and is caused by loss-of-function mutations in the dystonin gene (*DST*), which encodes a cytoskeletal cross-linking protein. Such mutations lead to microtubule instability. One clear difference between this mouse disorder and DYT1 dystonia is that the strong Golgi disorganization in the former leads to the neurodegeneration of sensory neurons of dorsal root ganglia. The lack of Golgi change in DYT1 dystonia is consistent with the observed lack of neurodegeneration.

Third, the Golgi apparatus can increase in size, which is the converse of the Golgi fragmentation discussed above. In mice lacking the gene encoding synaptotagmin IV (*Syt4*), a protein that is potentially involved in the transport of synaptic vesicles down the axons, the Golgi apparatus of hippocampal neurons becomes enlarged, with vesicles accumulating near the Golgi apparatus and the number of synaptic vesicles reduced, possibly as a consequence of defective axonal transport [112].

The lack of change in Golgi morphology in the context of the *Tor1a*^ΔE^ mutation is consistent with the finding that the mutation does not cause significant changes in neuronal levels of Golgi-associated protein, GM130.

### What does *Tor1a*^ΔE^ mutation influence in relation to the Golgi apparatus?

Golgi size is more likely to be regulated by the amount of transport cargo and activities of the Golgi-resident proteins than by mechanisms that regulate transport through the Golgi [113]. Thus the lack of change in Golgi size and shape does not necessarily indicate that Golgi-associated transport phenomena are intact. It is possible that an abnormality in Golgi-associated transport exists but is not sufficiently severe to change the Golgi structure at a level discernable by light microscopy. For example, concomitant changes in transport toward and away from the Golgi (e.g. both reduced or both enhanced) would lead to unchanged size at the steady-state level, yet might have an impact on cell function. In fact, torsinA has been reported to regulate the trafficking of some proteins to the plasma membrane, e.g. trafficking of the dopamine transporter, norepinephrine transporter, dopamine receptor, adrenergic receptor, and ATP-sensitive potassium channel [114]. This result could reflect an involvement of the Golgi, though this possibility has not been assessed directly. In addition, dendritic Golgi outposts are known to direct the growth of dendrites [11]. Thus, a subtle abnormality in Golgi function, if present, could underlie the subtle dendritic abnormality seen in neurons of HET mice [96, 109, 110].

In summary, our study demonstrates that the *Tor1a*^ΔE^ mutation does not affect the morphology of the neuronal Golgi apparatus. This result indicates that any changes in Golgi function that might be present are not severe enough to influence static Golgi morphology at a level detectable by light microscopy. Therefore, future studies should focus on more subtle and transient changes in Golgi function that are tangential to morphological changes, and on the features of other cellular organelles.

## Supporting information

S1 Fig Live-cell staining of the Golgi apparatus in cultured neurons using a fluorescent ceramide analogue. Pseudocolored, merged images corresponding to grayscale images shown in Fig 1. **A:** Color overlay of green band-pass and red long-pass images. The result demonstrates overwhelmingly strong, diffuse, non-specific staining in the green emission range of BODIPY FL C_5_-ceramide (Ceramide). The grayscale image with green long-pass emission filter (520-nm LP, Fig 1) is a correct representation of optical results. In contrast, the color overlay shown here is an attempt to reproduce the green long-pass image from its two components (green band-pass or red long-pass), but is not necessarily a correct representation. This is because 1) each grayscale image (green band-pass or red long-pass) was contrast-adjusted, such that the minimum and maximum in each image take values of 0 and 255 on an 8-bit intensity scale, and 2) the relative intensities of the two images are unknown. **B:** Ceramide staining (red) and nuclear staining (blue) in a single neuron.
(PDF)

S2 Fig Western blotting of GM130. This whole-membrane image corresponds to the data presented in Fig 14.
(PDF)
