# Supplementary material for: Structure of the Golgi apparatus is not influenced by a GAG deletion mutation in the dystonia-associated gene Tor1a
Source: PLoS One. 2018 Nov 7;13(11):e0206123. doi: 10.1371/journal.pone.0206123 (PMC6221310; doi:10.1371/journal.pone.0206123)
Supplement: S1 Fig — Pseudocolored, merged images corresponding to grayscale images shown in Fig 1. A: Color overlay of green band-pass and red long-pass images. The result demonstrates overwhelmingly strong, diffuse, non-specific staining in the green emission range of BODIPY FL C5-ceramide (Ceramide). The grayscale image with green long-pass emission filter (520-nm LP, Fig 1) is a correct representation of optical results. In contrast, the color overlay shown here is an attempt to reproduce the green long-pass image from its two components (green band-pass or red long-pass), but is not necessarily a correct representation. This is because 1) each grayscale image (green band-pass or red long-pass) was contrast-adjusted, such that the minimum and maximum in each image take values of 0 and 255 on an 8-bit intensity scale, and 2) the relative intensities of the two images are unknown. B: Ceramide staining (red) and nuclear staining (blue) in a single neuron. (PDF) [file pone.0206123.s001.pdf]

SUPPORTING INFORMATION (Mitchell et al.)

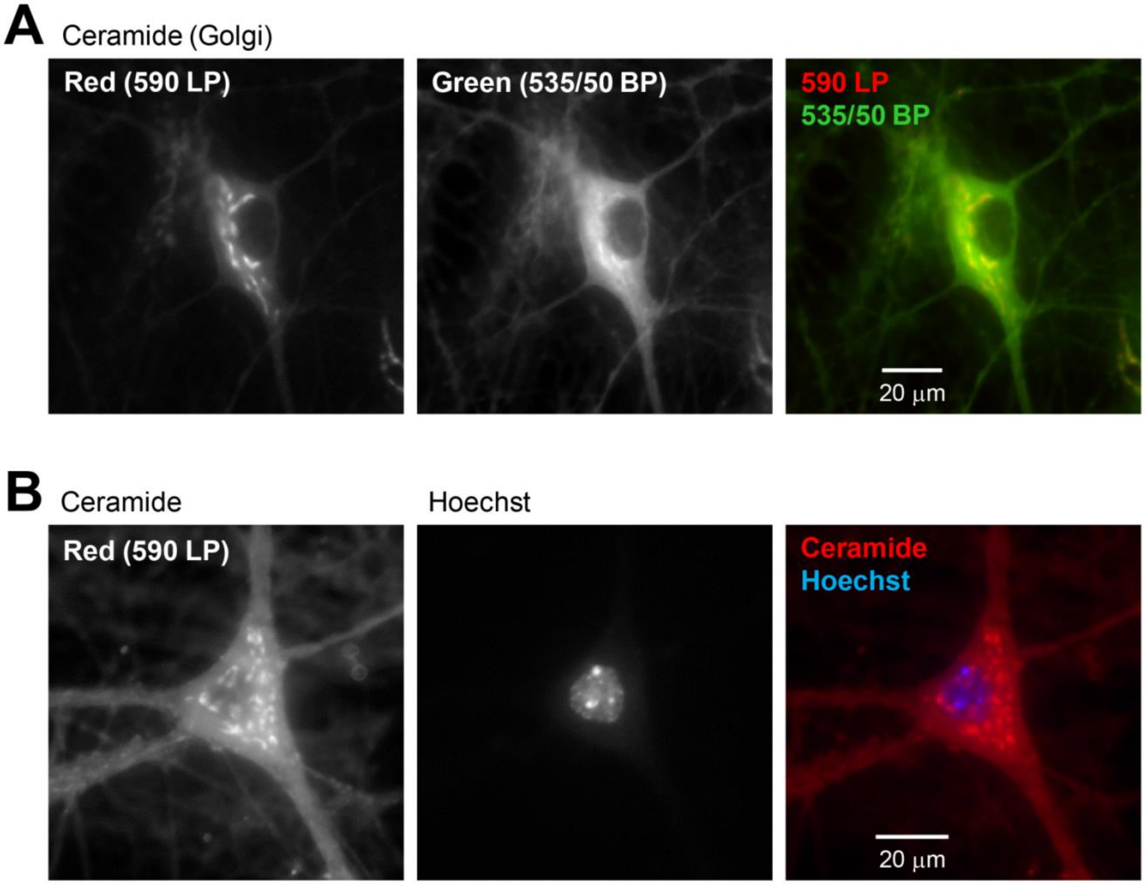

S1 Fig. Live-cell staining of the Golgi apparatus in cultured neurons using a fluorescent ceramide analogue. Pseudocolored, merged images corresponding to grayscale images shown in Fig 1. **A:** Color overlay of green band-pass and red long-pass images. The result demonstrates overwhelmingly strong, diffuse, non-specific staining in the green emission range of BODIPY FL C_5-ceramide (Ceramide). The grayscale image with green long-pass emission filter (520-nm LP, Fig 1) is a correct representation of optical results. In contrast, the color overlay shown here is an attempt to reproduce the green long-pass image from its two components (green band-pass or red long-pass), but is not necessarily a correct representation. This is because 1) each grayscale image (green band-pass or red long-pass) was contrast-adjusted, such that the minimum and maximum in each image take values of 0 and 255 on an 8-bit intensity scale, and 2) the relative intensities of the two images are unknown. **B:** Ceramide staining (red) and nuclear staining (blue) in a single neuron.
