# Supplementary figures and images for: Structure of the Golgi apparatus is not influenced by a GAG deletion mutation in the dystonia-associated gene Tor1a
Source: PLoS One. 2018 Nov 7;13(11):e0206123. doi: 10.1371/journal.pone.0206123 (PMC6221310; doi:10.1371/journal.pone.0206123)

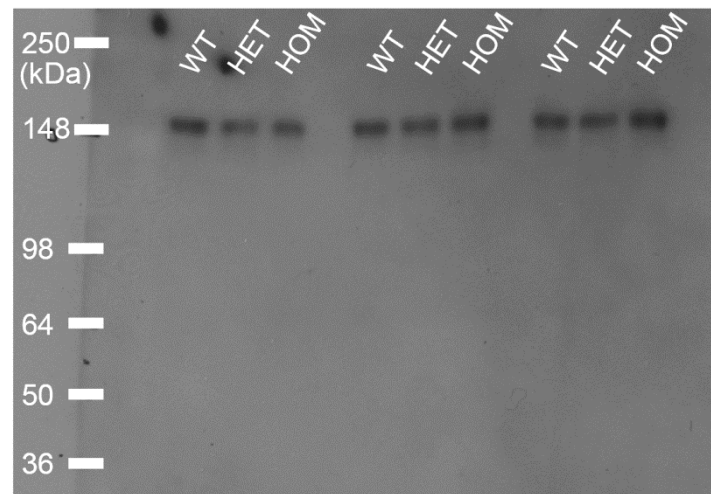

**S2 Fig. Western blotting of GM130.** This whole-membrane image corresponds to the data presented in Fig 14.

Supplement: S2 Fig — This whole-membrane image corresponds to the data presented in Fig 14. (PDF) [file pone.0206123.s002.pdf]
